# From Pharmacological Trigger to Manic State: Convergent Neurobiological Mechanisms of Drug-Induced Mania

**DOI:** 10.3390/brainsci16101081

**Published:** 2026-10-08

**Authors:** Ji Hyun Park

**Affiliations:** College of Pharmacy, Duksung Women’s University, 33 Samyang-ro 144-gil, Dobong-gu, Seoul 01369, Republic of Korea; pharmerpark@duksung.ac.kr

**Keywords:** circadian instability, drug-induced mania, glucocorticoid signaling, incentive sensitization, pharmacovigilance, secondary mania

## Abstract

Manic and hypomanic states are precipitated by pharmacologically diverse agents, including corticosteroids and antidepressants, and carry serious clinical consequences. Since the last cross-class review in 1995, the literature has fragmented into class-specific reviews lacking a shared mechanistic account. I hypothesize that chemically unrelated drugs perturb a few proximal systems—dopaminergic gain, glutamatergic synaptic potentiation, sleep and circadian timing, glucocorticoid signaling, and neuroimmune signaling—that converge on ventral prefrontal–amygdala–striatal circuitry implicated in secondary mania. Inverting the classical lesion-convergence logic, I treat pharmacological exposures as reversible, dose-graded probes of this network, accommodating withdrawal reversibility, dose dependence, and the affected minority. Evidence is appraised by study design rather than drug class, distinguishing experimentally supported associations from case series lacking exposure denominators. Across 15.4 million post-marketing reports containing 11,698 manic or hypomanic events, which cannot establish incidence or causality, median reported latency ranged from one day (glutamatergic agents) to three to four weeks (antidepressants), a contrast preserved at the drug level. A descriptive temporal-concentration flag identified signals warranting cautious interpretation. Rather than a universal pathway, this untested framework links proximal mechanism to phenotype and time course, generating testable predictions for studies combining exposure measurement, actigraphic sleep monitoring, and repeated activation ratings.

## 1. Introduction

### 1.1. Drug-Induced Mania as a Clinical and Scientific Problem

Mania and hypomania are states of abnormally and persistently elevated, expansive or irritable mood with increased energy or activity, accompanied by features such as decreased need for sleep, pressured speech, racing thoughts, distractibility and risk-taking behavior; a manic episode is, by definition, severe enough to cause marked impairment or to necessitate hospitalization [1]. Such states are not confined to primary mood disorders. They can be precipitated by pharmacological agents in wide use across medicine, among them systemic corticosteroids [2], antidepressants [3], psychostimulants [4], dopaminergic antiparkinsonian drugs [5], ketamine and esketamine [6], antibacterial agents [7] and tumor necrosis factor-α (TNF-α) inhibitors [8]. For some of these exposures, the association is quantitatively substantial: in a primary-care cohort of 372,696 patients, systemic glucocorticoid treatment was associated with a more than fourfold hazard of mania relative to unexposed patients with the same underlying diseases [9], and in adults with bipolar disorder, methylphenidate monotherapy was associated with a hazard ratio for mania of 6.7 within the first three months of treatment [10]. Because the implicated drugs are prescribed in nearly every specialty, often by clinicians who seldom encounter mania, a drug-induced manic state may go unrecognized, be attributed to a primary psychiatric illness, or be managed while the precipitating drug is continued. Recognizing the pharmacological precipitant matters both for immediate management—dose reduction, withdrawal or substitution—and for the diagnosis a patient subsequently carries, because the Diagnostic and Statistical Manual of Mental Disorders, fifth edition (DSM-5), distinguishes a substance/medication-induced bipolar and related disorder from an independent bipolar disorder [1].

Drug-induced mania also has a scientific significance that is distinct from its clinical implications. Clinically, a prescriber wants to know the risk attached to the drug in front of them, and a class-specific review serves that need. Scientifically, however, when a pharmacological exposure precipitates a manic state in a person with no previous episode, it is not merely a hazard to be catalogued; it also provides a perturbational probe of the systems involved in mania. Pharmacology here can play a role analogous to lesion neurology—altering or biasing a defined biological process and allowing the psychiatric consequence to be observed—with the additional advantages that exposure is dose-defined, graded, often reversible, and occasionally, repeated within the same individual. Treated as such, the drug literature constitutes a distributed set of probes into manic phenomenology, yet this perspective has received limited attention across pharmacological classes.

### 1.2. The Fragmentation of a Field

The last review devoted entirely to drug-induced mania as a single clinical entity, across all the chemical classes capable of producing it, was published in 1995. Peet and Peters [11] assembled the then-available case literature on corticosteroids, antidepressants, dopaminergic agents, thyroid hormone and isoniazid, and asked what these agents had in common, following the retrospective synthesis of Sultzer and Cummings [12]. Thirty-one years have since elapsed. Cross-class coverage has not vanished in that interval: mania appears as one entry among the medication-induced psychiatric disorders treated as a group [13,14], and manic and hypomanic states have been reviewed within a single clinical setting [15]. One later paper carries the cross-class title—Owley and Sharma [16], “Drug-induced mania: A critical review”—but its stated scope is antidepressant-associated mania in unipolar and bipolar affective disorder, and its organizing question is whether such episodes are drug-induced or expressions of the natural course of bipolar illness; it belongs to the switch literature rather than to the cross-class one, and is itself an instance of the nomenclatural slippage described in Section 3.1.1. In the literature identified by my search, I found no later synthesis that takes drug-induced mania itself as its subject and asks the comparative question across classes. The field has instead fissioned into single-class systematic reviews—of corticosteroids [2], TNF-α inhibitors [8], antibiotics in general [7] and clarithromycin in particular [17], atypical antipsychotics [18], antidepressants in bipolar depression [3], serotonergic psychedelics [19] and prescribed psychostimulants [4]. What is striking is how largely these studies have developed in parallel: they rarely cite one another, employ different case definitions and causality instruments, and seldom ask whether the manic states they describe might represent the same clinical endpoint reached through different pharmacological routes. As a result, the single-class review has become the field’s dominant unit of synthesis, leaving the cross-class question largely unaddressed.

The point was available from the beginning. Krauthammer and Klerman [20], in coining “secondary mania”, deliberately yoked pharmacological and structural etiologies into one category on the grounds that both demonstrated that a manic syndrome could arise without an antecedent affective diathesis. The two halves then had markedly different fates. The structural half matured into a sophisticated convergence literature [21,22,23,24]. The pharmacological half followed a different trajectory: it developed primarily through pharmacovigilance studies and single-class reviews, with comparatively little effort to integrate those findings into a broader mechanistic framework.

### 1.3. A Convergent Mechanistic Framework

That convergence literature supplies the endpoint of the framework proposed here. In pooled case material, focal lesions associated with secondary mania were predominantly right-hemispheric [21]; lesion network mapping has shown that anatomically heterogeneous mania-associated lesions share functional connectivity to a common network involving the right orbitofrontal cortex, right inferior temporal gyrus and right frontal pole [22]; and in longitudinally scanned bipolar patients, the communication of lesion-derived seeds with the amygdala and ventrolateral prefrontal cortex was specifically disrupted during the manic state [24]. Cotovio and Oliveira-Maia [23] integrated these findings into a convergent model of right ventral prefrontal hypoactivity with hyperactivity of the left amygdala, basal ganglia, and anterior cingulate cortex. The implicated ventral prefrontal–amygdala–striatal network therefore appears more closely related to the manic state than to a stable trait. What lesion mapping does not by itself reveal is how a system arrives at that state: it provides anatomical convergence without specifying a pharmacological route. This is the gap the present review addresses. I invert the lesion logic: rather than using lesions to localize mania, I use drugs as distributed pharmacological probes of the same network, asking which proximal mechanism each class most plausibly engages and how that perturbation could propagate into ventral prefrontal–limbic–striatal circuitry. The hypothesis developed in this review is that drug-induced mania may arise through a limited number of proximal mechanisms whose effects converge on a shared network, rather than through a collection of wholly agent-specific idiosyncratic reactions; the resulting convergence model is presented in Section 3.1.5.

Five proximal mechanisms are proposed. (i) Dopaminergic gain and incentive sensitization: agents that raise synaptic dopamine can precipitate elation, reduced need for sleep and goal-directed overactivity, repeated dopaminergic stimulation can progressively amplify incentive-motivational responses, and D2-family antagonists remain the most reliably antimanic drugs [25,26,27]. (ii) Glutamatergic signaling and rapid synaptic potentiation: subanesthetic N-methyl-D-aspartate (NMDA) receptor antagonists such as ketamine disinhibit cortical pyramidal neurons and initiate rapid, plasticity-dependent changes at excitatory synapses, the cascade thought to underlie their rapid antidepressant action and a plausible route to switching [28,29]. (iii) Sleep loss and circadian phase instability: sleep disturbance is the most consistent early symptom of mania, experimental sleep deprivation can itself precipitate switching, and in animal models, disruption of clock components that regulate midbrain dopamine produces mania-like behavior [30,31,32,33,34]; because many drug classes disturb sleep, this axis may also mediate the effects of the others. (iv) Glucocorticoid signaling: supraphysiological, glucocorticoid-receptor-weighted stimulation with suppression of endogenous cortisol alters the balance of limbic corticosteroid receptors, and glucocorticoids facilitate mesolimbic dopamine release in a state-dependent manner [35,36,37]. (v) Neuroimmune signaling: cytokine-based therapies and cytokine blockade perturb peripheral-to-central immune signaling, with the kynurenine pathway a candidate bridge to glutamatergic and dopaminergic function [38,39,40]. A pharmacokinetic gate, namely whether and to what degree an agent or its downstream signal reaches the brain, conditions all five. Each axis is developed in Section 3.3, together with what it fails to explain.

The five axes were selected by three criteria applied to the literature identified as described in Section 2.1, rather than defined in advance from receptor pharmacology. First, each is engaged by at least one drug class with human evidence of drug-associated manic states (Section 3.2): dopaminergic agents and psychostimulants for the first axis, ketamine and esketamine for the second, corticosteroids for the fourth, and interferons and TNF-α inhibitors for the fifth, while sleep curtailment is shared by several classes and can precipitate mania under experimental conditions [32,41]. Second, each has independent evidence, from studies of idiopathic mania or from experimental manipulation in humans or animals, that perturbing it produces manic or mania-like states rather than merely accompanying them. Third, each specifies a plausible route to the convergence network, and the axes differ in characteristic timescale, from hours for acute glutamatergic effects to weeks for monoaminergic adaptation, which makes them separable in principle in post-marketing latency data (Section 2.2). The partition is a working classification rather than an exhaustive taxonomy. The axes interact: glucocorticoids and sleep loss both modulate mesolimbic dopamine, and a single drug may engage more than one. Monoaminergic antidepressants, the most extensively studied precipitants, were not assigned a separate axis because serotonergic and noradrenergic reuptake inhibition do not by themselves specify a route to the manic state distinct from their downstream effects on dopaminergic transmission and sleep; in the convergence model, they are therefore mapped principally to dopaminergic gain, with a secondary contribution through sleep. Anabolic-androgenic steroids are considered alongside glucocorticoids as steroid-hormone exposures, although they act at a different receptor (Section 3.3.4), and a heterogeneous group of other central nervous system agents, such as levetiracetam, baclofen and zolpidem, is kept separate in the analyses rather than assigned to a single axis, although the convergence model indicates the axes they most plausibly engage.

### 1.4. Scope and Organization of the Review

The scope requires definition, since “drug-induced” is elastic. I include systemic pharmacological agents administered to humans for therapeutic purposes, together with experimental administration to volunteers where controlled data exist. I exclude substances used recreationally, for which exposure is often poorly quantified and polysubstance confounding is substantial; focal neuromodulation, admitted only as corroborating evidence where contact-level reversibility can adjudicate a claim pharmacology cannot; and mania secondary to medical illness, except where illness and treatment are difficult to separate.

**Figure 1:**
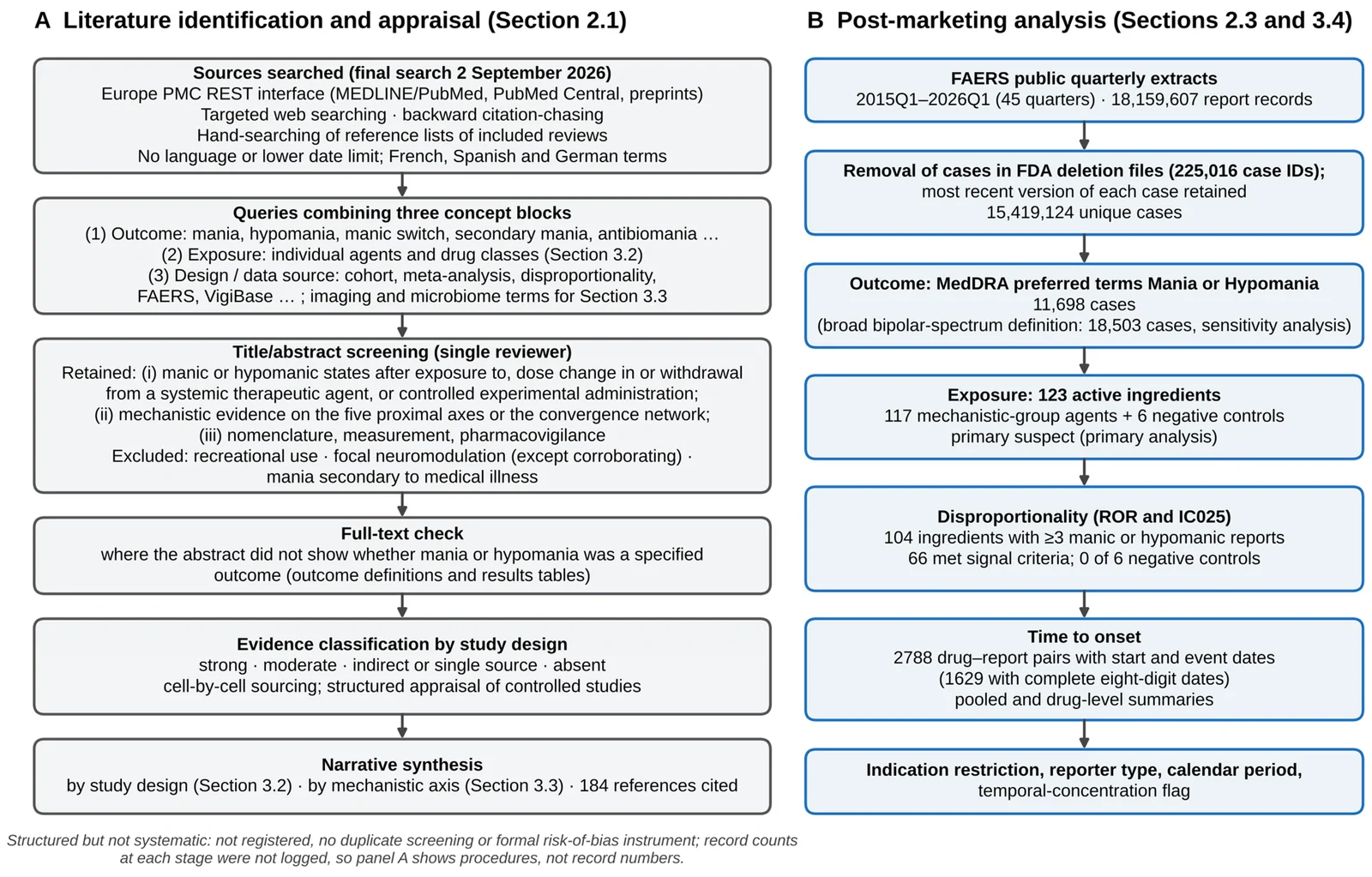
Flowchart of the review. (**A**) Identification, selection and appraisal of the literature (Section 2.1). The search was structured but not systematic; because record counts were not logged at each stage, the diagram shows the sequence of procedures rather than record numbers. (**B**) Construction of the post-marketing dataset and the analyses derived from it (Section 2.3 and Section 3.4). Search terms and eligibility criteria are given in Supplementary Table S6; the source of each evidence grade and the structured appraisal of controlled studies are given in Supplementary Tables S4 and S7; and the results of the post-marketing analysis are reported in Section 3.4 and Supplementary Tables S1–S3 and S5. FAERS, United States Food and Drug Administration Adverse Event Reporting System; IC025, lower bound of the 95% credibility interval of the information component; MedDRA, Medical Dictionary for Regulatory Activities; ROR, reporting odds ratio.

Section 2 describes how the literature was identified and appraised and how the post-marketing analysis was performed (Figure 1). Section 3.1 delimits the phenotype and argues that the field’s nomenclature is epistemically consequential; Section 3.2 maps the evidence by strength of attribution rather than volume of publication; Section 3.3 takes the five mechanistic axes in turn, assembles the convergence argument and specifies the pharmacokinetic gate; Section 3.4 reports an exploratory cross-class analysis of post-marketing reports; and Section 3.5 addresses susceptibility. Section 4 discusses what the analysis can and cannot establish, the mismatches between mechanism and data, and the clinical and research implications that follow.

## 2. Materials and Methods

### 2.1. Literature Identification and Evidence Appraisal

I searched Europe PMC—which indexes MEDLINE/PubMed, PubMed Central and preprint servers—through its REST interface, supplemented by targeted web searching, backward citation-chasing and hand-searching of the reference lists of every review that I included. The final search was run on 2 September 2026. Queries combined three concept blocks: outcome terms (mania, manic, hypomania, hypomanic, manic switch, secondary mania, antibiomania, bipolar disorder) applied to title and abstract fields; exposure terms covering the individual agents and drug classes treated in Section 3.2; and design and data-source terms (systematic review, meta-analysis, cohort, case–control, incidence, disproportionality, reporting odds ratio, pharmacovigilance, FAERS—the United States Food and Drug Administration Adverse Event Reporting System—VigiBase, EudraVigilance, the Japanese Adverse Drug Event Report database (JADER)), together with imaging and microbiome terms for the mechanistic sections. No language restriction or lower date limit was applied, and additional searches were conducted using French, Spanish and German terms. Where an abstract did not make clear whether a manic outcome had been pre-specified, I retrieved the full text and inspected the stated outcome definitions and results tables directly. The procedure is summarized in Figure 1A.

Records were screened by title and abstract, and full texts were examined when relevance or outcome definition could not be determined from the abstract. Sources were retained if they (i) reported manic or hypomanic states, or an outcome from which these could be separated, in association with exposure to, dose change in or withdrawal from a systemic therapeutic agent in humans, or with controlled experimental administration; (ii) addressed the neurobiology of one of the five proximal axes or of the network onto which they are proposed to converge, in humans or in experimental models; or (iii) provided context on nomenclature, measurement or pharmacovigilance methods. Recreational substance use, focal neuromodulation (except as corroborating evidence) and mania secondary to medical illness were excluded, as set out in Section 1.4. Because screening was performed by a single reviewer and record counts were not logged at each stage, Figure 1A depicts the sequence of procedures rather than the number of records at each step; 184 references are cited in the final synthesis. The search terms, sources and eligibility criteria are set out in full in Supplementary Table S6.

Two methodological points warrant clarification. First, the search for this narrative review was structured but was not designed as a systematic review: it was not registered, screening and extraction were performed without duplicate independent assessment, no formal risk-of-bias instrument was applied, and no attempt was made to retrieve the case-report literature exhaustively. Statements in this review regarding the absence of a particular study design should therefore be read as reporting the yield of the searches described here, not as claims of completeness. Because the evidence spans randomized trials, cohort and self-controlled studies, pharmacovigilance analyses, and case reports, no single risk-of-bias instrument could be applied across it. Three elements are provided instead to make the appraisal transparent: the design-specific grading described below; the source underlying every graded cell of the evidence-tier matrix (Supplementary Table S4); and a structured appraisal of the design, population, manic outcome ascertainment, and principal threats to validity of each controlled, cohort, self-controlled or pooled study that informs a strong or moderate grade (Supplementary Table S7). Second, evidence was graded using a four-level scheme applied throughout Section 3.2 and displayed in the evidence-tier matrix of that section. Each drug class was graded separately within each study design, so that each cell of the matrix describes the evidence available from that design, irrespective of the direction of its findings. Strong denotes two or more independent controlled, cohort or self-controlled studies, or a meta-analysis of such studies, in which mania or hypomania was a specified outcome; moderate denotes a single such study, or more than one controlled study in which manic or hypomanic events were systematically recorded but were not the specified outcome; indirect or single source denotes evidence bearing on a related phenotype or on a composite outcome from which mania cannot be separated, isolated uncontrolled observations, and all case-level evidence (case reports, case series and systematic reviews of case material) that lacks an exposure denominator and is therefore not graded above this level; and absent denotes that no evidence of that design was identified in the searches described above. The coding of each cell and the sources on which it rests are listed in Supplementary Table S4.

### 2.2. Rationale and Predictions for the Post-Marketing Analysis

The mechanistic framework developed in Section 3.3 generates several predictions that can be examined, albeit imperfectly, in post-marketing data. If pharmacologically triggered mania arises through a limited number of proximal mechanisms rather than through entirely agent-specific processes, reported events might show systematic differences in latency across mechanistic groups. The proposed axes operate on different characteristic timescales: acute glutamatergic effects occur within hours, sleep disruption and glucocorticoid effects over days, and some monoaminergic adaptations over longer intervals. Reported time to onset is not a direct measure of these mechanisms, but a reproducible ordering across drug groups would be compatible with the framework.

A second prediction concerns confounding by psychiatric indication. If an apparent association largely reflects the treatment of patients already at increased risk of mania or bipolar recurrence, excluding reports with recorded psychiatric indications should attenuate the disproportionality estimate. Conversely, persistence or strengthening of a signal after this restriction would be less consistent with indication alone as its explanation. Neither pattern is definitive: indication recording in spontaneous-report databases is incomplete, and residual confounding, differential reporting, and prescribing patterns remain possible.

I identified few previous disproportionality analyses in which mania was specified as an outcome of interest: studies have examined selective serotonin reuptake inhibitors [42], TNF-α inhibitors [43], intranasal esketamine [44], and pediatric antipsychotic withdrawal [45]. Mania and hypomania have also appeared as individual preferred terms in broader single-drug safety analyses in which they were not the primary focus [46,47,48]. In the literature identified by my search, I found no cross-class analysis comparing mechanistically distinct drug groups with mania or hypomania as the prespecified outcome.

I therefore performed an exploratory cross-class analysis of post-marketing reports to examine whether the mechanistic partition proposed in Section 3.3 was reflected in the reported latency and disproportionality patterns. The analysis was not designed to estimate incidence, absolute risk or causal effects for individual agents; its purpose was to examine whether routinely collected reporting data contained patterns compatible with, or contrary to, the proposed mechanistic framework.

### 2.3. Post-Marketing Data Source and Analysis

I used the public quarterly FAERS extracts released by the United States Food and Drug Administration (FDA) from 2015Q1 through 2026Q1. The FDA renamed the public reporting system the Adverse Event Monitoring System in March 2026; because the 2026Q1 extract retained the legacy FAERS file structure, I use “FAERS” throughout to refer to the data analyzed here. After the removal of cases listed in the FDA deletion files and retention of the most recent version of each case identifier, 15,419,124 unique cases remained (Figure 1B).

The primary outcome comprised the Medical Dictionary for Regulatory Activities (MedDRA) preferred terms Mania or Hypomania (11,698 cases). A broader bipolar-spectrum definition, comprising 18,503 cases, was examined as a sensitivity analysis. Exposure was defined at the active-ingredient level for 123 ingredients: 117 agents selected to populate the mechanistic groups examined in this review and six pharmacologically unrelated negative controls. Each case contributed at most once to a given ingredient, and the primary analysis was restricted to drugs recorded as the primary suspect.

Disproportionality was quantified using the reporting odds ratio (ROR) with its 95% confidence interval and the information component (IC) with its lower 95% credibility bound (IC025). A reporting signal was defined a priori as at least three cases, a lower 95% confidence bound for the ROR greater than 1, and an IC025 greater than 0. Secondary and sensitivity analyses broadened suspect-drug role and outcome definitions, excluded reports with bipolar-spectrum or other psychiatric indications, stratified by reporter qualification, calendar period and age, and examined reported time to onset from therapy initiation to the event, using drug–report pairs in which the ingredient was recorded as a primary or secondary suspect and both a therapy start date and an event date were available. Full methodological details and analysis code are provided in Supplementary Methods S1 and Code S1; results for all 123 ingredients across the primary and principal sensitivity analyses are reported in Supplementary Table S1, and the complete output of all fourteen analyses can be regenerated with Code S1. Reporting follows READUS-PV [49,50].

Generative artificial intelligence tools (Claude Opus 5, Anthropic, San Francisco, CA, USA) were used for English-language editing, code troubleshooting, and data visualization. They were not used to select analytical outcomes or interpret the findings. All analysis code was executed by the author, and all reported values and visualizations were reviewed by the author against the primary output files.

The interpretation of disproportionality estimates follows established pharmacovigilance principles [51,52]. An ROR describes the relative composition of a spontaneous-report database; it does not estimate incidence or clinical risk. Its magnitude can be influenced by prescribing patterns, indication, stimulated reporting, publicity, litigation, and the composition of the drug’s broader adverse-event report stream. I therefore use disproportionality here as a descriptive, hypothesis-refining measure to examine patterns generated by the mechanistic framework, not as evidence that an individual drug causes mania.

## 3. Results

### 3.1. The Phenotype and Its Boundaries

#### 3.1.1. Nomenclature and Its Incoherence

Several overlapping vocabularies are used to describe pharmacologically precipitated manic states, and they do not map neatly onto one another. The DSM-5 offers “substance/medication-induced bipolar and related disorder”, requiring that the disturbance develop during or soon after exposure, that the substance be capable of producing the symptoms, and—critically—that it not be better explained by an independent bipolar disorder [1]; the eleventh revision of the International Classification of Diseases (ICD-11) preserves a parallel structure [53]. The bipolar treatment literature uses “treatment-emergent affective switch” (TEAS), a term applied within populations with an established mood-disorder diathesis and therefore compatible with the drug acting as a precipitant rather than a sole cause. “Secondary mania” [20], in contrast, foregrounds an identifiable external or medical antecedent, whereas “antibiomania” [7] is primarily descriptive and carries no necessary implication about mechanism.

These terms are not synonymous; they define partly different case populations, and these differences directly affect case ascertainment and the interpretation of reported incidence estimates. A patient with bipolar I disorder who becomes manic on sertraline is a TEAS case, is largely excluded by DSM-5’s independence criterion, and would not have been called secondary mania. A rheumatology patient with no psychiatric history who becomes manic during infliximab treatment is a paradigmatic secondary-mania case but would generally fall outside the populations studied in the TEAS literature, which is largely based on bipolar cohorts. The antidepressant and TNF-α inhibitor literature therefore do not estimate directly comparable quantities, and their reported rates should not be interpreted as though they refer to the same underlying population or outcome definition. When I assert in Section 3.2 that the evidence base is weaker than its volume suggests, this is the first and most important reason.

#### 3.1.2. Phenomenology

Reported drug-associated manic states generally resemble recognized manic or hypomanic syndromes, although their ascertainment and clinical characterization vary considerably across drug classes. Three recurring features are worth highlighting.

First, the affective profile may differ by drug class. In the corticosteroid literature, irritability and dysphoric activation are prominent, psychotic features are common, and the classic euphoric presentation is not the modal one [2,54]; clarithromycin-associated episodes similarly carry a high burden of psychotic symptoms [17]. Whether this reflects a genuine mechanistic signature or ascertainment bias favoring more florid presentations is considered further in Section 3.1.4; the current data do not distinguish these explanations.

Second, dose dependence is documented most clearly for some classes. The Boston Collaborative Drug Surveillance Program [55] reported acute psychiatric reactions to prednisone increasing from roughly 1% below 40 mg daily to nearly 20% above 80 mg. Although these historical data concern psychiatric reactions broadly rather than mania specifically, they support an exposure–response relationship for corticosteroid-associated neuropsychiatric toxicity.

Third, a pre-existing psychiatric history is not a necessary feature of all reported drug-induced manic states: 93.2% of TNF-α inhibitor-associated cases had no history of psychiatric disorder or psychotropic treatment [8]. This argues against treating all drug-induced mania as the simple unmasking of latent bipolarity. The strongest evidence against that interpretation is experimental: Pope et al. [56], administering supraphysiologic testosterone to healthy men under randomized, placebo-controlled, crossover conditions, observed marked manic or hypomanic responses in a minority while most participants showed little change—and could not distinguish responders from non-responders on any baseline measure they collected. Susceptibility is real, then, but not reducible to detectable pre-existing affective illness. I take this up in Section 3.5.

#### 3.1.3. What Makes a Pharmacological Attribution Credible

Four features carry most of the weight in pharmacological attribution, and they are unequally satisfied. Temporal plausibility is the least discriminating and often the only feature available. Dose dependence provides stronger support where demonstrated and is best documented for corticosteroids and androgens. Dechallenge is commonly reported but is less specific than it may appear, because manic episodes can remit spontaneously and treatment is often changed simultaneously; improvement after drug withdrawal in a patient who also receives an antipsychotic therefore provides limited causal discrimination.

Rechallenge is the most discriminating of these features but is rarely available because deliberate re-exposure may be clinically or ethically unacceptable. Clarithromycin is the clearest pharmacological example, with rechallenge in five cases consistently reproducing the syndrome [17]. A complementary non-pharmacological example comes from Ulla et al. [57], who described hypomania in Parkinson’s disease that appeared and disappeared reproducibly when stimulation was switched between subthalamic contacts. This within-subject, spatially resolved reversal provides unusually strong corroborating evidence for a state-dependent perturbation and is therefore considered here despite deep brain stimulation falling outside the primary pharmacological scope.

#### 3.1.4. The Measurement Problem

The available instruments are poorly suited to this task. The Young Mania Rating Scale (YMRS) was developed to quantify manic symptom severity in patients with established mania [58], not to detect emergent activation in rheumatology or oncology outpatients, and no validated threshold exists for that purpose. MedDRA coding presents a different problem: “Mania” and “Hypomania” depend on reporter recognition and coding practice, whereas subsyndromal activation may instead be recorded under terms such as “Agitation”, “Insomnia”, “Euphoric mood”, or “Abnormal behavior”. Once distributed across such preferred terms, the phenotype is difficult to recover reliably through conventional signal-detection approaches. Adverse-event capture in randomized trials is often unstructured or dependent on spontaneous reporting, which is poorly suited to detecting a syndrome in which insight may be limited and early activation may not be experienced as problematic.

The resulting ascertainment biases are likely to operate differently across the severity spectrum. At the milder end, hypomanic and subsyndromal activation are likely to be under-ascertained because symptoms may not be elicited systematically, may not prompt spontaneous reporting, and may be distributed across several nonspecific adverse-event terms. At the severe end, causal attribution may instead be overconfident: a florid manic episode in a patient receiving several concomitant drugs may be attributed to a single suspected agent, with dechallenge interpreted as supporting causality despite concurrent treatment changes and the possibility of spontaneous remission. Incidence estimates in this literature should therefore be interpreted with both sources of ascertainment bias in mind.

#### 3.1.5. A Hypothesized Organizing Framework

**Figure 2:**
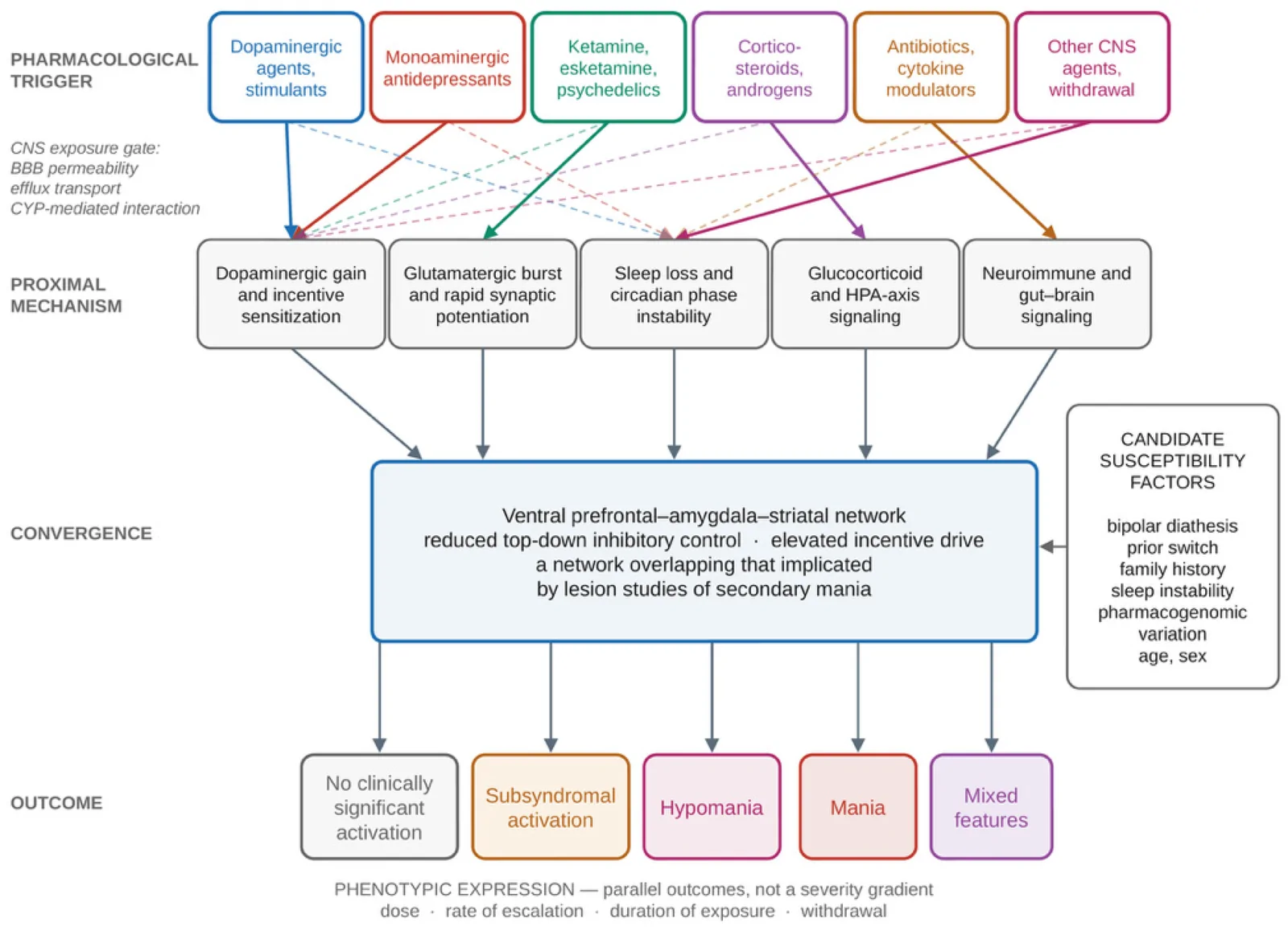
A hypothesized convergence model of drug-induced mania. Pharmacologically unrelated triggers (top) act on a small number of proximal mechanisms (second band), gated by whether and to what degree the agent reaches the central nervous system. Solid arrows denote the principal mechanism attributed to each class and dashed arrows a secondary contribution. The proximal mechanisms are proposed to converge on ventral prefrontal–amygdala–striatal circuitry, a network overlapping that is implicated by focal lesions causing secondary mania, where candidate susceptibility factors may determine whether the perturbation is expressed. Phenotypic expression is shown as parallel outcomes rather than as a single severity gradient: mixed features are not a more severe form of mania but a distinct presentation. Expression is modified by dose, rate of escalation, duration of exposure, and withdrawal. The framework is a hypothesis that has not been tested as a whole.

**Figure 3:**
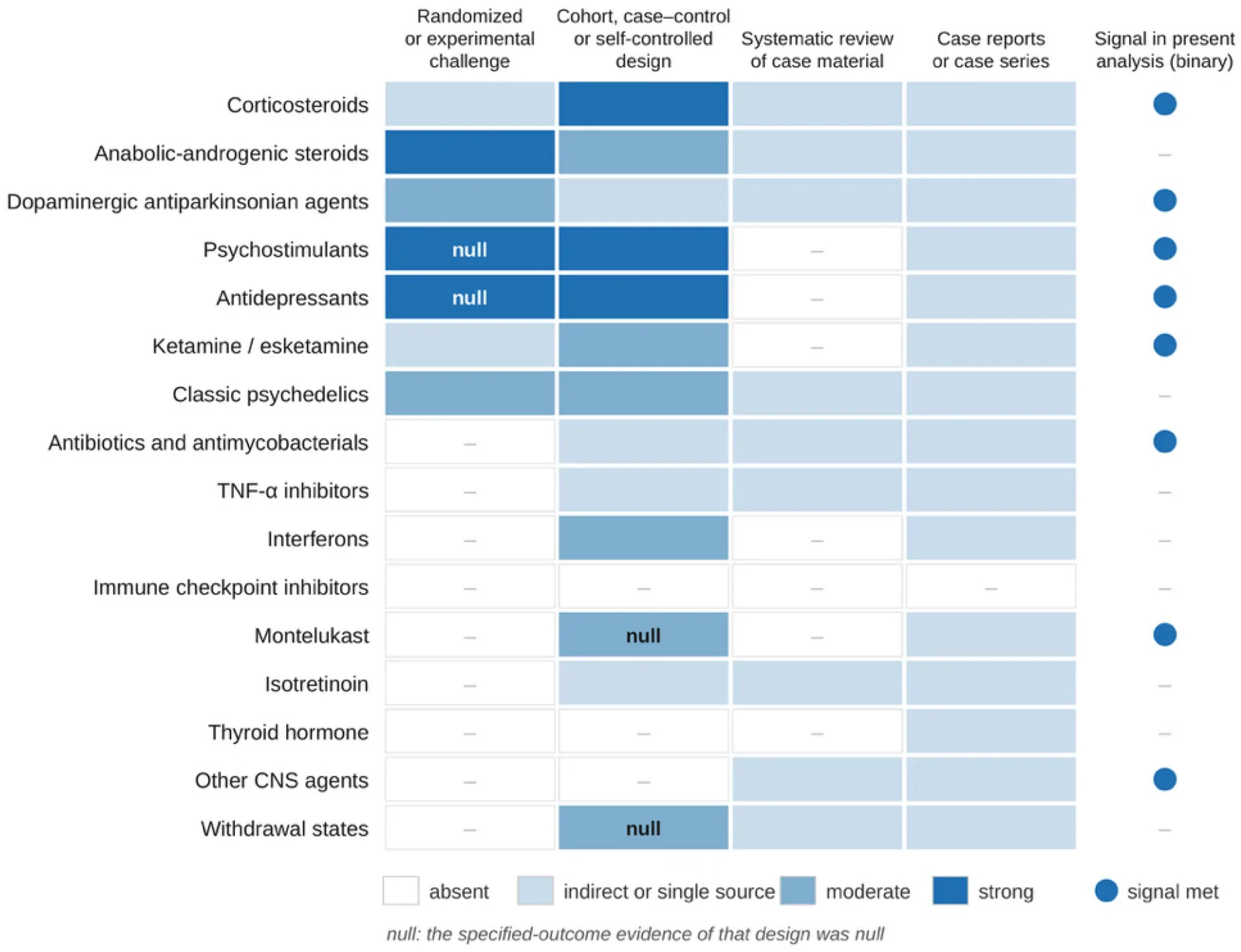
Evidence-tier matrix for drug classes associated with manic states. Each cell grades the evidence available from one study design for one class, using the definitions in Section 2.1: strong, two or more independent controlled, cohort or self-controlled studies, or a meta-analysis of such studies, with mania or hypomania as a specified outcome; moderate, a single such study, or more than one controlled study in which manic events were systematically recorded but were not the specified outcome; indirect or single source, evidence on a related phenotype or composite outcome, isolated uncontrolled observations, and all case-level evidence that lacks an exposure denominator and is therefore not graded above this level; absent (dash), no evidence of that design identified. Shading denotes the strength of the available evidence, not the direction of its findings; cells marked “null” are those in which the specified-outcome evidence did not show an excess of mania. The sources underlying each cell are listed in Supplementary Table S4. The rightmost column is a binary marker, not a shading level, and indicates only whether the agent or class met the signal criteria in the post-marketing analysis of Section 3.4. The matrix makes explicit that the classes for which mania is best documented clinically are not those for which it is best documented experimentally.

I can now state the organizing framework, which is offered as a hypothesis rather than as an established mechanism. Figure 2 presents the four-band model that structures the remainder of the review: a trigger band listing drug classes; a proximal mechanism band comprising the five axes; a convergence network band representing ventral prefrontal–amygdala–striatal circuitry implicated by the lesion literature; and an outcome band, the manic state. Two gates modulate flow between bands: central nervous system (CNS) exposure and individual susceptibility. Table 1 sets out the competing designations and what each includes and excludes, and Figure 3 grades each drug class not by publication volume but by the strength of the available study designs. Together, they make the review’s organizing argument visible in a single view: multiple proximal mechanisms may converge on a shared network, while the strength of evidence supporting individual drug–mechanism mappings remains highly uneven.

### 3.2. The Evidence Map Across Drug Classes

Reviews of drug-induced mania are conventionally organized by pharmacological class, an arrangement that can obscure substantial differences in the strength and type of evidence available for each class. The evidence ranges from randomized placebo-controlled crossover studies using validated mania scales to collections of published cases in which causal attribution was often based primarily on temporal association and dechallenge. A 4% rate observed in a randomized trial and an association inferred from a handful of published case reports represent fundamentally different levels of evidence and should not be interpreted as directly comparable. I therefore organize the evidence by study design rather than by receptor pharmacology, returning to the mechanism in Section 3.3. Table 2 provides the class-by-class summary, while Figure 3 presents the same evidence as a class-by-design matrix; the sections below focus on what each level of evidence can and cannot support.

#### 3.2.1. Classes with Experimental or Quasi-Experimental Evidence

Among the literature identified in my search, supraphysiologic androgen administration provides the clearest randomized, placebo-controlled evidence that a pharmacological exposure can precipitate hypomanic states in psychiatrically healthy volunteers, although the findings are not uniform. Pope et al. [56] gave testosterone cypionate in escalating doses to 600 mg per week against the placebo in a crossover trial of 56 men. YMRS scores rose significantly (*p* = 0.002), but the distribution matters more than the mean: of the 50 men receiving the full dose, 42 (84%) showed minimal effect, 6 (12%) became mildly hypomanic and 2 (4%) markedly so, and responders and nonresponders did not differ on any baseline demographic, psychological, laboratory, or physiological measure. Susceptibility was therefore not predictable from the baseline demographic, psychological, laboratory, or physiological measures collected in that study, and did not require a recognized bipolar disorder. Three additional controlled studies place this finding in context: a fixed-order crossover of methyltestosterone in 20 healthy men produced one manic and one hypomanic response [59]; randomization of 50 men to 100, 250 or 500 mg per week produced an agitated irritable manic syndrome in one participant at the highest dose [60]; and the same 600 mg weekly dose produced no significant mood effect in another study [61], while replacement-dose testosterone modestly improved mood, with mania neither assessed nor reported, in a large randomized trial in men with hypogonadism [62]. Taken together, the experimental evidence supports a dose-related effect at supraphysiologic exposure, but the finding has not been consistently reproduced. Observational evidence is consistent with this pattern: in a controlled comparison of 88 steroid-using and 68 non-using athletes, major mood syndromes, including mania and hypomania, occurred significantly more often during steroid exposure than in its absence and among nonusers [63], extending an earlier uncontrolled interview series of 41 users [64]. A systematic review of 37 studies of psychopathology in steroid-using athletes, eight of them case reports, likewise described mood destabilization, with hypomania predominating during short-term use [65]. The levodopa challenge of Murphy et al. [66] provides a second quasi-experimental strand—an early challenge experiment for which I identified no replication using modern instruments, and which should not be interpreted as an incidence estimate.

Antidepressants present a particularly informative discrepancy: randomized studies show little evidence of excess switching, whereas naturalistic studies report substantially higher rates. Oliva et al. [3], pooling 13 randomized trials and 1362 patients with bipolar depression, found no antidepressant significantly exceeding the placebo; venlafaxine carried the highest point estimate, with a risk ratio of 4.53, but the confidence interval was extremely wide (0.47–43.25), indicating substantial imprecision. A nationwide target trial emulation agreed, giving a hazard rate ratio for mania of 1.08 (0.72–1.61) with no separation by mood-stabilizer co-treatment [67]. Against this, Barbuti et al. [68] reported treatment-emergent mania or hypomania in 17.3% to 48.8% of exposed bipolar patients across 32 naturalistic studies. Trials generally enroll stabilized patients and observe them for relatively short periods, whereas naturalistic cohorts follow less selected populations for longer intervals and cannot fully separate treatment-emergent switching from the natural cyclicity of bipolar disorder. Randomized evidence provides a stronger basis for estimating treatment-attributable risk in stabilized bipolar patients, but it does not establish that all antidepressant-associated switching simply reflects unmasking of an underlying bipolar diathesis. Case reports also describe antidepressant-associated mania in a patient with no personal or family history of bipolar disorder who had no further mood episodes during two years of follow-up [69]. Evidence from other pharmacological classes likewise indicates that clinically recognized bipolar disorder is not a necessary precondition for every drug-associated manic state.

#### 3.2.2. Classes with Cohort or Self-Controlled Evidence

Systemic corticosteroids have one of the strongest observational evidence bases among the classes reviewed here. Fardet et al. [9] analyzed 786,868 courses in 372,696 primary-care patients and identified 10,220 incident severe neuropsychiatric events; against same-disease unexposed comparators, the hazard ratio (HR) for mania was 4.35, with a 95% confidence interval (CI) of 3.67 to 5.16, far above depression (1.83, 1.72–1.94). The higher relative risk for mania than for depression is particularly informative because it derives from denominator-based cohort data rather than from a selection of published cases. This pattern is consistent with the findings of Kusudo et al. [70], whose pooled analysis of 73 studies (*N* = 3,759,659) found manic symptoms to be more frequent than depressive symptoms within the first eight weeks, with an odds ratio (OR) of 2.42 (1.48–3.97). The classic dose–response figures of the Boston Collaborative Drug Surveillance Program [55], quoted in Section 3.1.2, must be flagged as secondary-sourced: they are quoted almost universally from later reviews, I could not verify them against the print original, and the numerator in the highest band is seven patients; the dose-related pattern they suggest, particularly above approximately 40 mg/day prednisone-equivalent, is also reflected in the later literature summarized by De Bock and Sienaert [2]. Onset is typically reported within one to two weeks, with improvement commonly described after dose reduction or withdrawal [71].

Psychostimulants provide the most direct, though not consistently replicated, evidence that concurrent treatment may modify risk. Viktorin et al. [10] followed 2307 adults with bipolar disorder initiating methylphenidate in Swedish registers using a within-individual design that controlled for stable individual characteristics, including genetic liability, although time-varying confounding cannot be excluded: on monotherapy, the hazard ratio for mania in the first three months was 6.7 (95% CI 2.0–22.4), whereas with a concurrent mood stabilizer, it was 0.6 (95% CI 0.4–0.9), with no evidence of an excess manic hazard in that stratum and a point estimate below unity. Replication has been inconsistent: a Danish mirror-image registry study of 1043 patients found fewer manic episodes after methylphenidate initiation, with or without mood stabilizers [72], and in a French claims-database replication, the excess observed without mood stabilizers disappeared in self-controlled case-crossover analyses [73]. Chiorean et al. [4], pooling five randomized studies (*n* = 1653), found no effect on manic symptoms. These findings are not necessarily inconsistent, because the randomized studies enrolled bipolar patients almost all already stabilized, precisely the stratum in which Viktorin found no excess risk. The randomized literature has therefore provided limited information on methylphenidate monotherapy in patients not protected by concurrent mood-stabilizing treatment. A related gap deserves plain statement: many adult studies pool mania with psychosis into a composite outcome [74,75,76], while studies that isolate mania do so in populations already carrying a bipolar diagnosis [10,73], so the incidence of stimulant-precipitated first-episode mania in adults without bipolar disorder remains unquantified. Dopaminergic antiparkinsonian therapy is quantified chiefly through impulse-control and dopamine dysregulation phenotypes rather than mania as such, a distinction worth preserving: an impulse-control disorder was present in 13.6% of 3090 treated patients, rising to 17.1% among dopamine-agonist recipients against 6.9% among non-recipients [5].

Ketamine and esketamine yield two counterintuitive findings. Liu et al. [77], in a propensity-matched cohort of 2126 patients with bipolar depression receiving esketamine plus a mood stabilizer, found no increase in manic switch and a significantly lower hazard at 1–180 days (HR 0.643, 0.442–0.935); confounding by indication and treatment selection cannot be excluded; one possible explanation is that effective treatment of the depressive episode reduces the clinical context in which switching is observed. Santucci et al. [78] reported a potentially mechanistically informative temporal pattern: across 45 patients and 518 patient-months, there were no manic or hypomanic events during acute twice-weekly induction, whereas 13 out of 45 patients (28.9%) had an episode during maintenance. This temporal pattern does not support the simple clustering of switches around acute induction and is compatible with risk emerging during repeated exposure; however, an uncontrolled cohort cannot establish whether cumulative exposure, illness course, or another time-varying factor accounts for the difference. The quantified cohorts identified here were drawn from bipolar populations, so their denominators are diathesis-positive by design.

#### 3.2.3. Classes Resting on Systematic Reviews of Case Material

For many agents, the best available synthesis is a systematic review of published cases, which can document reported occurrences and characterize features such as latency, duration and dechallenge, but cannot provide an incidence or risk estimate.

Antibiomania is the exemplar. Lambrichts et al. [7] identified 47 published cases implicating 12 antibacterial agents, most often antitubercular drugs, macrolides and quinolones; Marković et al. [17] narrowed the focus to clarithromycin, retrieving 34 patients whose episodes frequently carried psychotic features. The temporal signature is unusually tight: onset approximately four days after initiation, duration approximately three days, and full recovery in every patient after discontinuation, with recurrence reported in all five patients who were rechallenged, providing unusually strong case-level support for causal attribution. Yet no prospective study has estimated the incidence of mania during antibiotic therapy, and the denominator-based evidence is indirect: adolescent doxycycline exposure was associated with later manic-episode or bipolar diagnosis in a Swedish total-population cohort (adjusted hazard ratio 1.95, 1.49–2.55), an association the authors judged non-causal because comparable associations arose with other antibiotic classes [79]. The case literature supports causal attribution in selected individual patients, particularly where rechallenge is documented, but the population-level incidence of antibiotic-associated mania remains unknown [80].

Tumor necrosis factor-α inhibitors occupy a similar tier with one partial denominator. Miola et al. [8] retained four case reports plus a single cohort in which 40 out of 7600 infliximab-treated patients (0.53%) experienced elated mood episodes. Across the pooled sample of 44 patients, 97.7% met the criteria for mania rather than hypomania, and 93.2% had no reported history of psychiatric disorder or psychotropic treatment, indicating that documented pre-existing psychiatric illness is not a necessary feature of reported TNF-α inhibitor-associated manic states. For interferon-α, the case literature is supplemented by one prospective cohort in which manic and hypomanic states were systematically assessed [38] (Section 3.3.5), and mania has been reported both on exposure and on withdrawal [81].

Classic psychedelics have recently acquired a systematic review with meta-analysis: Eskinazi et al. [19] reported rates of hypomania or mania ranging from 5.8% in controlled trials of psilocybin-assisted psychotherapy for major depression to 30% in naturalistic studies of individuals with bipolar disorder. The marked difference between supervised trials and naturalistic bipolar samples is clinically informative, but the estimates are not directly comparable because the populations, screening procedures, and exposure settings differ substantially. Outside supervised settings, a systematic review of case reports identified 17 affective episodes, including mania, after psychedelic use in individuals without a previous major psychiatric disorder [82], and mania has been described after psilocybin ingestion in a woman without a prior bipolar diagnosis [83]. The evidence for herbal agents is particularly limited: 35 case reports across 11 herbal medicines, only one applying a standardized causality algorithm and only one assaying the preparation—which proved adulterated with a pharmaceutical that plausibly accounted for the reaction [84].

#### 3.2.4. Classes Where the Evidence Is Weaker than Its Reputation

Several agents commonly listed as potentially manicogenic have substantially less mania-specific evidence than such listings may imply.

Levetiracetam illustrates this problem clearly. Stoppa et al. [85] reassessed 50 published cases of levetiracetam-associated psychiatric episodes and found that only eight were (hypo)manic, the remaining 42 being psychotic, with formal Naranjo appraisal [86] indicating a low probability that levetiracetam alone was responsible. Thus, the number of published cases specifically meeting a manic or hypomanic phenotype is much smaller than the broader psychiatric case literature suggests, and formal causality appraisal further weakens attribution to levetiracetam alone.

Montelukast presents a different problem: much of the controlled psychiatric evidence is reassuring but is not mania-specific. A nationwide Swedish active-comparator cohort of 74,291 children found a weighted hazard ratio of 0.99 (95% CI 0.84–1.16) for a psychiatric composite that did not include mania [87]. One controlled study has specified a manic outcome: in a propensity-matched cohort of 154,946 patients, Paljarvi et al. [88] included incident manic episode or bipolar disorder among their pre-specified diagnoses and reported no excess. A null result for a broad psychiatric composite should therefore be distinguished from the much more limited evidence addressing mania specifically; the latter now includes at least one controlled cohort with a prespecified manic outcome. At the case level, hypomania was reported one week after montelukast was added in an older woman with long-remitted bipolar disorder, resolving after discontinuation [89].

Isotretinoin has a substantial quantitative psychiatric literature that does not address mania, concerning suicide, depression, and aggregate psychiatric morbidity, heavily confounded by acne severity and largely null in the best-controlled designs [90]. Mania as a distinct outcome remains supported mainly by case reports, such as psychotic mania within the first week of treatment in a young man without a personal or family psychiatric history [91], and by a structured review of this case literature concluding that many isotretinoin-associated states recorded as depressive had manic or mixed features [92], while two larger studies addressed related psychiatric outcomes without providing a mania-specific risk estimate. A global cohort study of psychiatric disturbance in isotretinoin users does not separate manic outcomes [93]. A twenty-year disproportionality analysis of psychiatric adverse events reported for the drug does isolate Mania as a preferred term, 48 out of the 254 reports composing its bipolar-disorder category, alongside Bipolar disorder (167), Bipolar I disorder (25), Flight of ideas (10), and Bipolar II disorder (4), with hypomania absent—but it estimates disproportionality for that composite category rather than for mania, and reports no mania-specific point estimate or interval [94]. My own analysis returned 22 manic or hypomanic reports for isotretinoin among 34,801, a reporting odds ratio of 0.83 (95% CI 0.55–1.27), meeting no signal criterion. Mania is therefore identifiable in these datasets, but has rarely been analyzed as a distinct outcome with its own effect estimate.

Varenicline occupies an intermediate position that is easy to misstate in either direction. Case reports describe mania or hypomania during treatment, in patients with established bipolar disorder [95,96,97], in patients with no psychiatric history [98], as late-life first-onset mania [99,100], and once following cessation rather than initiation [101]. I identified no case series of three or more patients, cohort study or systematic review, and one report argues the opposite direction [102]. In the reporting analysis of Section 3.4, varenicline met the signal criteria (reporting odds ratio 4.01, 95% CI 3.06–5.25; 53 reports; Supplementary Table S1), which is consistent with the case literature and shares its limitations. Taken together, these findings constitute convergent but low-tier evidence for an association, rather than evidence sufficient to establish risk. Thyroid hormone is similarly limited to case reports after levothyroxine replacement [103] and older descriptions of mania in thyrotoxicosis, several involving lithium-induced thyroid dysfunction in patients already carrying a bipolar diagnosis, leaving causal direction unresolvable.

Immune checkpoint inhibitors, finally, are better characterized as an evidence gap than as an established association with mania. Despite strong a priori immunological plausibility and an extensive literature on other neurological and psychiatric immune-related adverse events, I found no case report, case series, cohort or systematic review of checkpoint-inhibitor-induced mania; the only quantitative resource is a broad disproportionality analysis of psychiatric disorders that does not isolate mania [104]. This absence should be interpreted as a gap in mania-specific study rather than as evidence that checkpoint inhibitors do or do not carry such a risk.

#### 3.2.5. Withdrawal- and Discontinuation-Emergent Mania

Mania has also been reported following drug withdrawal or discontinuation—reported after the discontinuation of antidepressants [105], cannabis, lithium, interferon-α and stimulants—but it is almost entirely case-report-based. More importantly, the methodologically strongest study of withdrawal-emergent neuropsychiatric outcomes returned a negative result for mania: in a self-controlled case series of 21,995 patients discontinuing long-term glucocorticoid therapy, Fardet et al. [106] found significant elevations for delirium or confusion (incidence rate ratio 2.67, 1.96–3.63) and marginally for depression, but none for mania—despite mania being a pre-specified outcome, and despite the same investigators’ fourfold manic hazard during exposure in the same data source. Thus, although glucocorticoid withdrawal was associated with some neuropsychiatric outcomes, the strongest denominator-based study identified here did not show an increased incidence of mania following discontinuation.

The classes surveyed here share no single obvious pharmacological mechanism. An androgen receptor agonist, a glucocorticoid, a dopamine reuptake inhibitor, a macrolide antibiotic, a monoclonal antibody against TNF-α, a serotonin 2A (5-HT2A) receptor agonist and an NMDA receptor antagonist have no common molecular target, transporter or metabolic pathway, and several are not centrally acting in any conventional sense. That such pharmacologically heterogeneous exposures have each been associated with a broadly recognizable manic phenotype raises the question of whether their effects can be understood at a level of organization above the individual receptor or molecular target.

### 3.3. Convergent Neurobiological Mechanisms

The classes surveyed in Section 3.2 have no common molecular target. What follows develops the hypothesis that they nonetheless share a small set of proximal effects, and that each class can be read as a probe of one of them. Five axes are treated in turn, followed by the network onto which they converge and the pharmacokinetic gate determining whether a systemically administered agent reaches it. Each subsection closes by stating what that axis, considered as a probe, reveals about the manic state and what it fails to explain. The criteria by which these axes were selected are set out in Section 1.3.

#### 3.3.1. Dopaminergic Gain and Incentive Sensitization

Mania has been attributed to excessive dopaminergic transmission for half a century, but the hypothesis was born of pharmacology rather than of measurement. Agents that raise synaptic dopamine precipitate elation, pressured speech, reduced sleep need, and goal-directed overactivity, while D2-family blockers remain the most reliably antimanic compounds available, outperforming anticonvulsants at the acute manic endpoint in both major quantitative syntheses [25,26]. D2-family receptor antagonism or partial agonism is a common pharmacological feature of effective antipsychotic treatments for acute mania, supporting a causal role for dopaminergic signaling in the manic state. This remains, however, a reversal argument: therapeutic efficacy does not establish dopaminergic excess as the primary origin of mania, any more than aspirin’s efficacy establishes prostaglandin excess as the origin of a headache.

**Human Molecular Imaging Evidence**

Ashok et al. [107] formalized this intuition into a two-state model in which mania reflects elevated striatal D2/D3 availability with a hyperactive reward network, but the imaging evidence is thinner than the model implies. Elevated striatal D2 binding was reported with a high-affinity tracer, yet only in psychotic bipolar patients and in proportion to psychosis rather than to mood severity [108], and elevated presynaptic synthesis capacity in first-episode bipolar psychosis was also observed in schizophrenia [109], suggesting that this finding may index psychosis more broadly rather than mania specifically. The most state-related finding is lower striatal dopamine transporter binding in bipolar disorder (by 22–24%), most pronounced in current mania, with values closer to those of the controls in a separate group of recently remitted patients [110]. This is compatible with altered dopaminergic regulation, but transporter binding does not directly measure synaptic dopamine clearance, the design was cross-sectional, with only nine patients in current mania, and the finding has not, to my knowledge, been independently replicated. Particularly informative is the finding that amphetamine challenge in euthymic bipolar patients produced no greater tracer displacement than in the controls despite a significantly greater behavioral response [111]. This dissociation suggests that heightened behavioral responsivity need not reflect greater presynaptic dopamine release and may instead involve postsynaptic or circuit-level sensitivity.

**Incentive Sensitization and the Parkinsonian Natural Experiment**

Behavioral sensitization converts a transient pharmacodynamic event into a persistent state: progressive amplification of the incentive-motivational response to a fixed dose across intermittent exposures, with wanting dissociating from liking [27]. One molecular mediator implicated in behavioral sensitization is ΔFosB, which accumulates in D1-expressing striatal neurons and is associated with enduring changes in glutamatergic synaptic function [112]. These findings provide a preclinical mechanism by which repeated dopaminergic stimulation could produce effects that outlast acute drug exposure, but extending this mechanism directly to mania remains a translational hypothesis.

Parkinson’s disease provides a clinically informative natural experiment in chronic dopaminergic overstimulation. Dopamine dysregulation syndrome—compulsive overuse of short-acting levodopa or apomorphine, with hypomanic “on” states and dysphoric “off” crashes—arose, across 98 published cases, mostly in patients without prior substance-use or psychiatric history [113]; affected patients show enhanced levodopa-induced ventral striatal dopamine release tracking compulsive wanting but not liking [114], the incentive-salience dissociation the theory predicts. Impulse-control disorders form a second phenotype and are more strongly associated with dopamine agonists, particularly those with relative D3 preference, than with levodopa [5]. Because D3 receptors are enriched in ventral striatal circuitry, receptor selectivity may contribute to the phenotypic distinction between impulse-control disorders and dopamine dysregulation syndrome.

**Why Only a Minority Switch**

Dopaminergic exposure is common and drug-induced mania rare. Candidate-gene associations involving COMT or DRD2/DRD3 have not yielded robust, consistently replicated signals at a genome-wide scale; current evidence instead supports a polygenic architecture, with DRD2 among loci implicated in well-powered bipolar disorder studies [115]. A candidate clinical moderator is pharmacological rather than genetic: among the 2307 bipolar adults initiating methylphenidate, the hazard of mania within three months was 6.7 (95% CI 2.0–22.4) on monotherapy but 0.6 (0.4–0.9) with concomitant mood-stabilizing treatment [10], although this pattern was not reproduced in Danish registry data [72].

Considered as a pharmacological probe, this axis supports the possibility that increased incentive salience and ventral striatal responsivity contribute to manic activation. The clinical and translational evidence further suggests that dose, temporal pattern of stimulation and concurrent mood-stabilizing treatment may modify this response, although these factors have not been tested within a single mechanistic model. What this axis does not explain is equally important: dopaminergic gain alone does not readily account for reduced need for sleep, grandiosity, or the temporal course of a manic episode. Dopaminergic gain is therefore better viewed as one input to a vulnerable network than as a sufficient explanation for the full manic syndrome.

#### 3.3.2. Glutamatergic Signaling and Rapid Synaptic Potentiation

**The Disinhibition Cascade**

A widely studied model of rapid-acting glutamatergic antidepressant action comprises an experimentally dissociable cascade, several steps of which are potential points at which manic switching could theoretically emerge. In preclinical models, subanesthetic ketamine preferentially reduces NMDA-receptor-mediated activity in fast-spiking parvalbumin interneurons, disinhibiting pyramidal neurons and increasing cortical glutamatergic output [28]. Human magnetic resonance spectroscopy studies provide partial support for an acute increase in prefrontal glutamatergic activity, including changes in glutamate plus glutamine (Glx) and glutamate–glutamine cycling [116]. In preclinical models, downstream α-amino-3-hydroxy-5-methyl-4-isoxazolepropionic acid (AMPA)-receptor activation is required for ketamine-like behavioral effects, while reduced spontaneous NMDA-receptor signaling has been linked to the rapid dendritic translation of brain-derived neurotrophic factor (BDNF) and subsequent tropomyosin receptor kinase B (TrkB)–mechanistic target of rapamycin complex 1 (mTORC1) signaling, with associated increases in synaptic spine formation [29,117,118].

**The Paradox: One Cascade, Two Directions**

Several properties of this cascade that are relevant to rapid antidepressant action also make it a plausible candidate mechanism for manic switching, although this argument is mechanistic rather than directly empirical. Unlike conventional monoaminergic antidepressants, ketamine can alter affective state within hours and rapidly modifies excitatory synaptic function. This raises the possibility that in susceptible individuals, the same plasticity-promoting processes that contribute to antidepressant effects could also facilitate excessive activation. Whether failure of homeostatic synaptic scaling contributes to such switching remains hypothetical—more so because the machinery ketamine transiently disables is the machinery implementing that scaling [29]. The cascade may also intersect with the dopaminergic axis: prefrontal glutamatergic activation can increase excitatory drive to the ventral tegmental area and alter striatal dopamine release [119], providing one plausible point of convergence between glutamatergic and dopaminergic mechanisms. The translational specificity of these models is limited, however, because ketamine-based paradigms have been used to model both mania-like activation and psychosis-related phenotypes [120].

**Clinical Switch Data: Timing Rather than Agent**

Pooled trial data in bipolar depression give a low but non-zero switch rate, six out of 235 participants (2%) across eight studies [6], and the two cohorts described in Section 3.2.2 sharpen the picture. Esketamine under mood-stabilizer cover carried no increase in switch at any interval [77], whereas in routine care, no patient switched during acute twice-weekly induction while 13 out of 45 (28.9%) did so during maintenance [78]. If switching is driven primarily by acute NMDA-receptor blockade or the immediate glutamatergic response, one might expect events to cluster around individual administrations. Instead, events in this cohort occurred during maintenance treatment, a pattern compatible with cumulative or time-dependent processes, including but not limited to synaptic plasticity or sensitization. An uncontrolled cohort, however, cannot distinguish among these mechanisms. Case reports extend the observation beyond bipolar illness, describing a switch into mania during subanesthetic ketamine treatment of unipolar treatment-resistant depression [121]. Dextromethorphan–bupropion and 5-HT2A agonists have also been linked experimentally to plasticity-related and synaptogenic pathways despite acting through different proximal targets [122], although switch evidence for both remains limited to case reports.

Considered as a probe, this axis reveals that mania can be entered from the cortical end of the network by potentiating the very synapses whose hypofunction is thought to sustain depression, and that entry is a function of cumulative rather than instantaneous exposure. What it fails to explain is why the same intervention yields remission in most recipients and elevation in a few. Controlled data on ketamine or esketamine without concurrent mood-stabilizing treatment are extremely limited, leaving uncertainty about whether mood-stabilizer co-treatment materially modifies switch risk in this setting.

#### 3.3.3. Sleep Loss and Circadian Phase Instability as a Final Common Pathway

**The Most Consistent Immediate Antecedent**

Wehr et al. [30] proposed that the precipitants of mania share one downstream property—the capacity to curtail sleep—and that because mania itself is often accompanied by a markedly reduced need for sleep, the process could become self-amplifying once initiated. Descriptive evidence is broadly consistent across methods: sleep disturbance was the most robust early symptom of mania across 17 prodrome studies with a median prevalence of 77% [31]; actigraphy showed most patients passing through one or more 48-h sleep–wake cycles when switching out of depression [32]; and among the 3140 interviewed patients, sleep loss as a self-reported trigger of high mood was associated with bipolar I subtype (OR 2.81, 95% CI 2.26–3.50), while equivalent analyses for low mood were null [123]. This asymmetry is compatible with a model in which sleep loss contributes preferentially to manic rather than depressive destabilization. Prospective confirmation is nevertheless thinner than the retrospective literature implies: the most-cited prospective test found that sleep deficit predicted depressive symptoms but not mania over six months [124].

**Experimental Deprivation and the Chronotherapeutic Paradox**

The causal claim rests on experimental induction. Of the nine rapid-cycling patients kept awake for 40 h, eight switched out of depression and seven were rated manic or hypomanic [32]—a striking experimental observation, but one that requires caution because the sample was small, unblinded, and selected for rapid cycling. In 206 unselected bipolar depressed patients given three cycles of total sleep deprivation, the switch rate was 4.85% into mania and 5.83% into hypomania [41]—the order of magnitude of antidepressant-associated switching, where no agent significantly exceeds the placebo [3]. The paradox is that the same manipulation is a rapid antidepressant, with over half of the depressed patients responding to one night of deprivation and 83% of unmedicated responders relapsing after a single night of recovery sleep [125]. Bright light is similarly double-edged yet shows no clear switch signal in modern protocols [126]. These findings illustrate that the same circadian intervention can be associated with antidepressant benefit in most patients and manic switching in a minority, with dose, timing, duration, and concurrent pharmacotherapy among the plausible modifiers of that divergence.

**Mechanism: The Clock Transcriptionally Gates Dopamine**

Acute sleep loss engages precisely the circuitry of Section 3.3.1 and Section 3.3.2: one night of deprivation reduces striatal [^11^C]raclopride binding, consistent with increased dopamine release [127], amplifies mesolimbic reward reactivity [128], and alters prefrontal–amygdala functional coupling [129], affecting circuitry that overlaps components of the network discussed in Section 3.3.6 despite the absence of a direct pharmacological receptor target. At the molecular level, the clock gates dopamine transcriptionally. ClockΔ19 mice show a mania-like syndrome of hyperactivity, decreased sleep, and heightened reward value for cocaine and sucrose, normalized by chronic lithium and rescued by viral re-expression of functional CLOCK in the ventral tegmental area alone [33]; REV-ERBα represses tyrosine hydroxylase transcription in midbrain dopamine neurons, so that its deletion raises dopamine and produces mania-like behavior [34]; and circadian-inappropriate daytime spikes in dopaminergic activity drive rapid mood cycling [130], suggesting in these models that the timing of dopaminergic activity may be as important as its aggregate level. The translational relevance of these models remains limited: no corresponding single-gene CLOCK abnormality has been established as a cause of human mania. The models therefore demonstrate that perturbing clock–dopamine coupling can generate mania-like phenotypes, not that this mechanism explains the etiology of human mania.

**Do the Other Drug Classes Act Through Sleep?**

Sleep differs from the other proposed axes because it may function not only as an independent mechanism, but also as a mediator of their effects. Corticosteroids suppress and fragment rapid eye movement (REM) sleep [131]; methylphenidate raised the pooled risk of insomnia across 35 blinded placebo-controlled trials [132], and in one adult with attention-deficit/hyperactivity disorder, actigraphy recorded shorter and poorer sleep preceding a manic episode after methylphenidate initiation [133]; wake-promoting agents are efficacious in bipolar depression but carry treatment-emergent hypomania [134]; many antidepressants alter sleep architecture, including the suppression of REM sleep [135]. If several chemically unrelated drug classes alter sleep, and sleep loss independently perturbs dopaminergic and prefrontal–limbic function, then sleep disturbance could represent one route through which otherwise distinct pharmacological mechanisms converge. In this sense, it is a candidate final common pathway rather than simply a parallel mechanism. The claim must be made carefully, because it is untested: no study formally models drug exposure → objective sleep change → mood outcome as a mediation, and the sharpest counterexample is esketamine, associated with treatment-emergent insomnia yet showing no increased switch risk under mood-stabilizer cover [77]. Interventional data are correspondingly split: enforced darkness accelerated symptom decline in acute mania only within two weeks of onset [136]—a narrow window consistent with the self-amplification model of Wehr et al. [30].

Considered as a probe, sleep and circadian manipulation show that manic activation can occur without the direct engagement of a single pharmacological receptor target and raise the possibility of an early period during which destabilization remains reversible before a more persistent state is established. What this axis fails to explain is specificity—sleep loss is ubiquitous, mania rare—and it cannot yet be shown to mediate any drug’s effect.

#### 3.3.4. Glucocorticoid Signaling and the Hypothalamic–Pituitary–Adrenal Axis

**Dose–Response, Timing and Polarity**

Exogenous glucocorticoids are among the best quantitatively characterized pharmacological precipitants of manic symptoms, and their principal signaling system—glucocorticoid and mineralocorticoid receptor regulation within the hypothalamic–pituitary–adrenal (HPA) axis—has been studied directly in mania. The dose–response and hazard data are set out in Section 3.1.2 and Section 3.2.2; three features of their pattern constrain mechanism. Onset is early and dose-tracking. The predominant affective presentation also appears to vary with duration, with activation, euphoria and hypomanic symptoms reported more often early in treatment and depressive symptoms more often during prolonged exposure. Prior reactions have not proved reliably predictive of response to a later course [137], suggesting that susceptibility is not fully captured by stable clinical characteristics alone.

**Glucocorticoid Receptor Overstimulation with Mineralocorticoid Receptor Deprivation**

The mineralocorticoid receptor (MR) has higher affinity for endogenous glucocorticoids and is substantially occupied at basal concentrations, particularly in limbic regions, whereas the lower-affinity glucocorticoid receptor (GR) is recruited more strongly as glucocorticoid concentrations rise during circadian peaks and stress [35]. Many synthetic glucocorticoids favor GR over MR signaling to varying degrees and suppress adrenocorticotropic hormone (ACTH) secretion, thereby reducing endogenous cortisol, an important physiological ligand for central MR. This combination of exogenous GR stimulation, variable MR activity and suppression of endogenous cortisol creates a receptor-signaling profile that differs from physiological stress responses. An altered MR/GR balance is therefore a plausible contributor to corticosteroid-associated mood activation, but its role in human steroid-induced mania has not been directly tested. One test of this hypothesis would be whether partial restoration of MR signaling modifies steroid-associated activation; to my knowledge, such an intervention has not been evaluated clinically. The same framework could in principle generate distinct neuropsychiatric effects during withdrawal as endogenous HPA signaling recovers; however, the strongest denominator-based study of glucocorticoid discontinuation did not detect an increased incidence of mania [106].

**Glucocorticoid Potentiation of Mesolimbic Dopamine**

One plausible route linking glucocorticoid exposure specifically to behavioral activation is through the modulation of mesolimbic dopamine, providing a point of interaction between the mechanisms discussed in Section 3.3.1 and Section 3.3.4. Corticosterone at stress-range concentrations is intrinsically reinforcing [138], and its dopaminergic effect is state-dependent, raising accumbal dopamine only during the active phase and above a threshold concentration [36,37], indicating that glucocorticoid effects on mesolimbic dopamine can depend on physiological state and circadian timing, thereby linking this axis conceptually to Section 3.3.3. Preclinical work provides a candidate circuit: prefrontal glucocorticoid receptors can modulate stress-evoked dopamine efflux through glutamatergic projections influencing the ventral tegmental area [139]. In humans, amphetamine-induced striatal dopamine release has been associated with individual cortisol responses to psychosocial stress [140], supporting physiological coupling between HPA and dopaminergic systems. I identified no study directly imaging striatal dopamine release during corticosteroid-induced hypomania.

**Hypothalamic–Pituitary–Adrenal Findings in Bipolar Disorder, and Two Comparator Exposures**

HPA dysregulation is present in acute mania and partially normalizes in remission [141], and enhanced ACTH response to corticotropin-releasing hormone during euthymia was associated prospectively with subsequent mania [142], suggesting that HPA hyper-reactivity may contribute to vulnerability to manic recurrence rather than merely reflecting the acute mood state. Meta-analysis of 41 studies confirmed elevated basal and post-dexamethasone cortisol and ACTH, greatest in the manic phase, yet concluded that HPA dysregulation is not an endophenotype [143].

An endogenous comparator helps to frame the pharmacology. In Cushing’s syndrome, 25 out of 30 patients met the criteria for an affective episode, but 20 for depression and only 8 for mania or hypomania [144]—a predominance of depression that contrasts with the activation, euphoria, and hypomanic symptoms reported early in therapeutic glucocorticoid treatment. The two exposures differ in ways that could plausibly account for this: endogenous hypercortisolemia is chronic, gradual in onset, and engages both MR and GR over prolonged periods, whereas therapeutic synthetic glucocorticoids are typically introduced abruptly, at supraphysiological doses, on a non-physiological schedule, and with GR-weighted receptor activity. The contrast therefore raises the possibility that rate of onset, receptor selectivity, and circadian pattern of exposure contribute to affective polarity, but the two settings differ in many other respects and do not permit those factors to be isolated causally. Anabolic-androgenic steroids form a distinct route, acting at the androgen receptor and through aromatization rather than at GR, yet converging phenomenologically; they provide the clearest randomized evidence identified in this review that a pharmacological exposure can induce hypomanic symptoms in psychiatrically healthy volunteers (Section 3.2.1).

Considered as a pharmacological probe, glucocorticoid exposure is unusually informative because it provides denominator-based risk estimates, evidence of dose-related neuropsychiatric effects, and a clinically apparent dependence on timing and duration. Preclinical work further offers plausible links from cortical glucocorticoid signaling through glutamatergic pathways to mesolimbic dopamine, connecting this axis to the mechanisms discussed in Section 3.3.1, Section 3.3.2 and Section 3.3.3. These links remain mechanistic hypotheses rather than a fully demonstrated pathway for corticosteroid-induced mania. What this axis still fails to explain is individual susceptibility. Population-level dose–response relationships coexist with substantial person-to-person heterogeneity, and prior reactions do not reliably predict the response to subsequent exposure. The androgen literature shows a similar pattern under more controlled conditions: group-level effects are detectable, yet individual responders remain difficult to identify prospectively.

#### 3.3.5. Neuroimmune Signaling and the Gut–Brain Route

Peripheral immune abnormalities are well-documented in bipolar disorder, but evidence for an immune signature specific to mania is considerably less consistent and is more directly relevant to a mechanism of manic activation. Pro-inflammatory and regulatory cytokines rise together, indicating a dysregulated set-point rather than a discrete insult [145], and no single analyte discriminates mood phase across 53 studies [146]. C-reactive protein (CRP) is the exception, elevated in bipolar depression and euthymia but more substantially in mania and falling more after a manic episode [147]—though it does not track symptom severity, and the sleep loss and hyperactivity of mania can themselves raise it. Dispersion may be as informative as the mean: variability relative to controls is increased for CRP, interleukin (IL)-6, and TNF-α during mania [148], suggesting substantial heterogeneity in inflammatory activity among manic patients. Such heterogeneity is compatible with, but does not establish, the possibility that immune signaling contributes more strongly in a susceptible subgroup.

Interferon-α provides one of the clearest human models of cytokine-associated psychiatric change. Its predominant affective effect is depressive: across 26 prospective studies, the cumulative incidence of major depressive episodes reached 0.25 at 24 weeks [149]. Manic and hypomanic states nevertheless occur, and their reported profile is informative: among the 93 interferon-naive patients followed prospectively, 30 (32%) developed a mood disturbance, of which frank mania accounted for 3, irritable hypomania for 15, and depressive mixed states for 12 [38]. Interferon-associated mood disturbance thus appears to span manic and depressive phenomenology rather than presenting predominantly as euphoric mania, which reframes the question from whether a drug causes mania to whether it destabilizes mood along a mixed-state axis. Mania has also been reported around dose reductions, treatment interruptions, and withdrawal [81], raising the possibility that rapid changes in interferon signaling, rather than sustained cytokine exposure alone, may contribute in some cases.

The converse possibility—that cytokine blockade can precipitate mood elevation—is supported by substantially more limited evidence. The only systematic review retained four single-patient reports and one uncontrolled cohort in which 40 out of 7600 infliximab-treated patients (0.53%) experienced elated mood [8]; pharmacovigilance adds 41 reports in the bipolar-psychotic spectrum among the 9942 TNF-inhibitor adverse-reaction reports, with improvement on dechallenge in 22 out of 23 evaluable patients [43]. Rechallenge data are absent, and patients starting biologics are usually tapering corticosteroids at the same time. Meanwhile the same intervention is under development for the opposite pole, its antidepressant effect concentrated in high-CRP patients [150] and absent in bipolar depression [151]. Two explanations reconcile the paradox. One possibility is that the rate of change in immune signaling matters in addition to its absolute level. Reports of mania around interferon withdrawal and after TNF blockade are compatible with such a model, although existing data are insufficient to establish a common mechanism. A second possibility involves TNF–type I interferon cross-regulation: anti-TNF treatment can prolong type I interferon production by plasmacytoid dendritic cells by altering their maturation [152,153]. This provides a testable hypothesis by which interferon-associated and TNF-inhibitor-associated mood activation could share part of the same signaling pathway, but the connection has not been demonstrated clinically.

“Antibiomania”, named by Abouesh et al. [154] from spontaneous reports dominated by clarithromycin, ciprofloxacin, and isoniazid, is routinely offered as evidence for a gut–brain route. Its anchor is Yolken et al. [155]: recent antimicrobial exposure was over-represented among the 234 patients hospitalized for acute mania (adjusted OR 5.5, 95% CI 2.2–14.1) but not in other psychiatric admissions—a design that cannot separate antibiotic-induced mania from infection-induced mania with the antibiotic as its marker. Microbiome evidence proper amounts to one unreplicated trial in which probiotics reduced rehospitalization after mania [156]. Three non-microbiome mechanisms also warrant consideration: fluoroquinolones are γ-aminobutyric acid type A (GABA-A) receptor ligands [157]; clarithromycin inhibits CYP3A4 and P-glycoprotein, potentially increasing exposure to co-prescribed corticosteroids and psychotropics (Section 3.3.7); and clarithromycin can itself produce neurotoxicity with limbic encephalitis-like presentations [158]. These mechanisms are at least as plausible as a gut–brain explanation for the available case literature for three reasons: the approximately four-day median onset with recovery after discontinuation [17], which is temporally compatible with accumulating enzyme inhibition; the concentration of reported cases among agents with independent central pharmacology rather than simply among broad-spectrum antibiotics expected to produce substantial microbiome disruption; and the occurrence of positive rechallenge. No human study has measured the microbiome after antibiotic exposure and before incident mania.

The kynurenine pathway provides one plausible molecular bridge between immune activation and the glutamatergic and dopaminergic systems implicated in mania, although findings differ across biological compartments. The kynurenic-to-quinolinic acid ratio is decreased in mood disorders [39], whereas cerebrospinal fluid kynurenic acid is elevated in bipolar disorder and was higher in euthymic patients who had experienced mania within the preceding year [40,159]. In addition, kynurenine metabolites, but not cytokines, distinguished acutely manic patients from the controls [160]. Taken together, these findings support the kynurenine pathway as a candidate mediator, but neither the direction of its effect nor its causal role in mania has been established. More broadly, peripheral immune signaling remains a plausible and partly characterized route to manic activation, while the central mechanisms linking peripheral immune changes to the manic state remain unproven in humans.

#### 3.3.6. Convergence onto a Lesion-Defined Network

Krauthammer and Klerman [20] coined “secondary mania” for manic syndromes following physical illness or drugs, placing the structural and the pharmacological under one causal heading—a framing this review inherits. The lesion literature that followed has consistently suggested a right-hemisphere predominance in secondary mania, with earlier work also proposing a lateralized contrast between left anterior lesions associated with depression and right-sided, limbic-connected lesions associated with mania. Pooling 114 reports, Barahona-Corrêa et al. [21] found that of the 201 patients with focal lesions, 60.7% had lesions confined to the right hemisphere against 11.4% to the left, though published lesion images were, as they caution, selected for interest; and mania occurred in 6 out of 66 prospectively followed head-injury patients, predicted only by basal temporal polar lesions and unrelated to psychiatric family history [161].

The central problem of this literature is that mania-associated lesions are anatomically heterogeneous. Cotovio et al. [22] addressed this heterogeneity using lesion network mapping, computing for each of the 41 mania-associated lesion locations the network functionally connected to it in a normative connectome of 1000 healthy individuals, comparing these with 79 non-mania lesions, and replicating the analysis in two independent cohorts. Lesion locations were heterogeneous, with no region lesioned in all or even most cases, arguing against a simple model in which mania localizes to a single anatomical “center”; instead, the lesions shared a connectivity profile involving the right orbitofrontal cortex, right inferior temporal gyrus, and right frontal pole, which was stable across normative connectomes [162]. Lee et al. [24] added a bridge to idiopathic illness: seeds derived from 23 mania-causing lesions showed, in longitudinally scanned bipolar patients, specific disruption of their communication with the amygdala and ventrolateral prefrontal cortex during the manic state. Cotovio and Oliveira-Maia [23] synthesized both studies into a convergent model—hypoactivity of right ventral prefrontal cortex with hyperactivity of left amygdala, basal ganglia, and anterior cingulate cortex, with lateralized asymmetry providing a particularly testable feature of the model.

Two additional lines of evidence implicate overlapping circuitry. Functional imaging shows failure to activate right orbitofrontal cortex during a Go-NoGo task in mania [163], with meta-analytic evidence suggesting that at least some abnormalities are state- or task-dependent rather than simply reflecting a shifted resting baseline [164]. This raises the possibility that pharmacological triggers might also be expressed through altered task-dependent responsivity. Left ventrolateral prefrontal activity during reward expectancy prospectively predicts mania risk a year later [165]. Stimulation is the sharper line, being reversible, gradable, and site-selectable as lesions are not: deep brain stimulation produces contact-dependent, reproducible hypomania, one electrode giving different mood states from different contacts [57], localizing to the medial forebrain bundle rather than the subthalamic nucleus itself [166]. The reversibility and contact specificity of stimulation-induced hypomania provide unusually strong within-subject evidence linking perturbation of this circuitry to mood state.

Here the review’s own argument enters. As a heuristic, I propose that a systemically administered manicogenic drug can be viewed as a distributed lesion: not a structural injury to the nodes of the mania network, but a pharmacological perturbation of the neuromodulatory inputs that regulate their activity. The nodes—right orbitofrontal, inferior temporal and basotemporal cortex, frontal pole, caudate, thalamus, amygdala, ventral striatum—are the targets of no drug in Section 3.2. What such agents alter is the gain of ascending dopaminergic, noradrenergic, serotonergic, and cholinergic systems innervating those nodes unequally, Coenen’s medial forebrain bundle interpretation of stimulation-induced hypomania [166] being the structural statement of this claim. This framing offers a potential explanation for three features of drug-induced mania that are less readily accommodated by a focal-lesion model. First, it is compatible with reversibility, because the pharmacological modulation of network inputs is potentially reversible when exposure is reduced or withdrawn, unlike a fixed structural lesion. Second, it is compatible with dose dependence, because the magnitude of neuromodulatory perturbation can vary continuously with exposure. Third, it offers a framework for the rarity of the response: if pharmacological exposure perturbs network inputs but clinical expression depends on the state of the recipient network, identical exposures could produce different outcomes across individuals. In principle, such susceptibility could become measurable, but the relevant network-level predictors have not yet been established. The model may also help reconcile the weak association between lesional mania and psychiatric family history with the stronger role of pre-existing vulnerability in some forms of drug-associated switching. A structural lesion may impose a network perturbation largely independent of baseline susceptibility, whereas a smaller pharmacological perturbation might be more dependent on the pre-existing state of the network. This remains a hypothesis rather than a demonstrated distinction.

I wish to be precise about what is claimed. The network is Cotovio’s, as is the demonstration that heterogeneous lesions converge on one reproducible connectivity profile; this review offers no rival model and no additional nodes, its contribution being the inverse mapping—reading the pharmacology through that network. The framework is interpretive rather than tested: no study has compared the connectivity of pharmacologically triggered mania against the lesion-derived network, and that drugs act on inputs rather than nodes is an inference from receptor distribution, not a demonstrated fact. A direct test of this framework—resting-state imaging during corticosteroid- or dopamine-agonist-induced mania, seeded on the Cotovio regions and repeated on remission—is now feasible.

#### 3.3.7. The Pharmacokinetic Gate: CNS Exposure, Transporters, and Drug–Drug Interactions

Each mechanistic account above depends on a pharmacokinetic prerequisite: the relevant drug, metabolite, or peripheral signal must reach its target at sufficient effective exposure. The governing quantity is neither plasma nor total brain concentration but the unbound brain-to-unbound-plasma ratio, Kp,uu, where unity indicates passive equilibration and values below unity net efflux [167]; exposure is also regionally heterogeneous [168], which is the pharmacokinetic mechanism by which a systemically administered drug can act like the focal perturbation of Section 3.3.6. For many substrates, active efflux transporters—particularly P-glycoprotein—are important determinants of Kp,uu below unity. Clarithromycin inhibits both CYP3A4 and P-glycoprotein [169], creating the potential for increased systemic exposure, and for susceptible transporter substrates, altered brain exposure; the magnitude of either effect is drug- and dose-dependent. ABCB1 polymorphisms have also been associated with antidepressant outcomes in patients receiving P-glycoprotein substrate antidepressants [170], but these findings have not entered routine pharmacogenetic guidance.

Agents with minimal blood–brain barrier penetration can nevertheless influence central signaling indirectly. Monoclonal antibodies typically achieve brain concentrations of only about 0.1% of plasma [171], making substantial direct CNS receptor occupancy by TNF-α inhibitors an unlikely primary mechanism, despite reports of mania with improvement after dechallenge [43]. Several peripheral-to-central signaling routes could account for such effects without substantial antibody penetration: cytokines can cross via saturable transport [172] and signal at the endothelium, which functions as an interface rather than a simple barrier [173]; vagal paraganglia can respond rapidly to IL-1 receptor ligands without requiring blood–brain barrier transit [174]; and inflammation can alter blood–brain barrier permeability [175]. Montelukast is the instructive intermediate: over twelve weeks, it reached only very low cerebrospinal fluid (CSF) concentrations yet lowered CSF inflammatory markers and [^11^C]PBR28 binding [176], while its epidemiological signal is for anxiety and insomnia rather than mania [88].

In the absence of a concurrent effect on transport or binding, metabolic inhibition need not alter Kp,uu itself; however, increased unbound systemic exposure will still increase unbound brain concentration proportionally when Kp,uu remains constant. Clarithromycin provides a useful pharmacokinetic example: as a mechanism-based CYP3A4 inhibitor, its inhibitory effect develops over several days and can persist after discontinuation [177], a time course compatible with the approximately four-day median onset reported for clarithromycin-associated mania. Several published cases also involved concomitant corticosteroid use [178], providing a plausible setting in which a pharmacokinetic interaction could contribute to the observed neuropsychiatric event. In cases involving concomitant corticosteroids or other CYP3A4 substrates, a pharmacokinetic interaction is therefore a plausible alternative or contributing explanation and should be considered before attributing the episode solely to clarithromycin. Interindividual metabolic variation provides a related source of exposure heterogeneity. Escitalopram exposure is 2.63-fold higher in CYP2C19 poor metabolizers [179], and antidepressant response overlaps pharmacogenomically with treatment-emergent mania [180].

Some reactions labeled “idiosyncratic” may instead reflect unmeasured variability in pharmacokinetic exposure. If typical brain exposure lies below the concentration required for a relevant target effect, individuals at the upper end of the exposure distribution may be disproportionately represented among those who react, while genetic variation, enzyme inhibition and organ impairment can further widen between-patient differences in exposure. Some apparently agent-specific cases of mania may therefore reflect pharmacokinetic or pharmacodynamic interactions that are difficult to disentangle in spontaneous reports, because suspect designation reflects the reporter’s attribution and detailed information on drug concentrations and interacting exposures is rarely available. Consistent with this limitation, TNF inhibitors were the sole suspected drug in only 56% of the reports analyzed by Yelnik et al. [43]. I identified no published case of drug-induced mania in which the measured plasma or cerebrospinal fluid concentrations of the implicated agent were used to support causal attribution.

### 3.4. Post-Marketing Reporting Across Drug Classes

**Figure 4:**
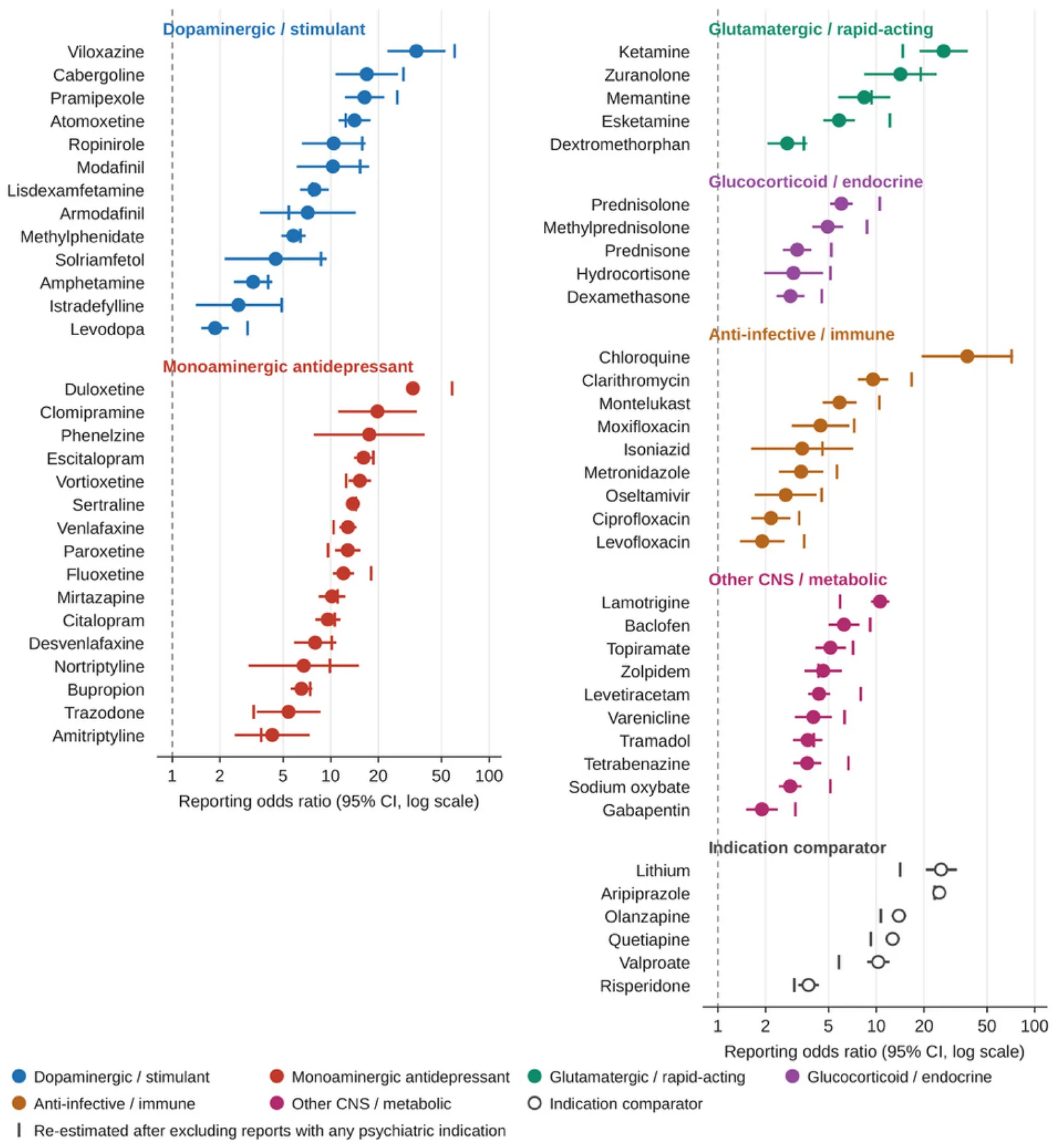
Reporting odds ratios for mania or hypomania in the United States Food and Drug Administration Adverse Event Reporting System, 2015Q1–2026Q1, for agents meeting the signal criteria with at least five reports, grouped by the mechanistic groups used in Section 3.4. Circles denote the reporting odds ratio with a 95% confidence interval in the primary analysis (drug recorded as primary suspect); vertical ticks denote the estimate re-computed after excluding all reports carrying a psychiatric indication. Open circles are psychotropic agents included as indication comparators. Negative control agents, none of which produced a signal, are omitted and are given in full in Table 3. Reporting odds ratios describe disproportionality within spontaneous reports and are not measures of incidence or risk.

**Figure 5:**
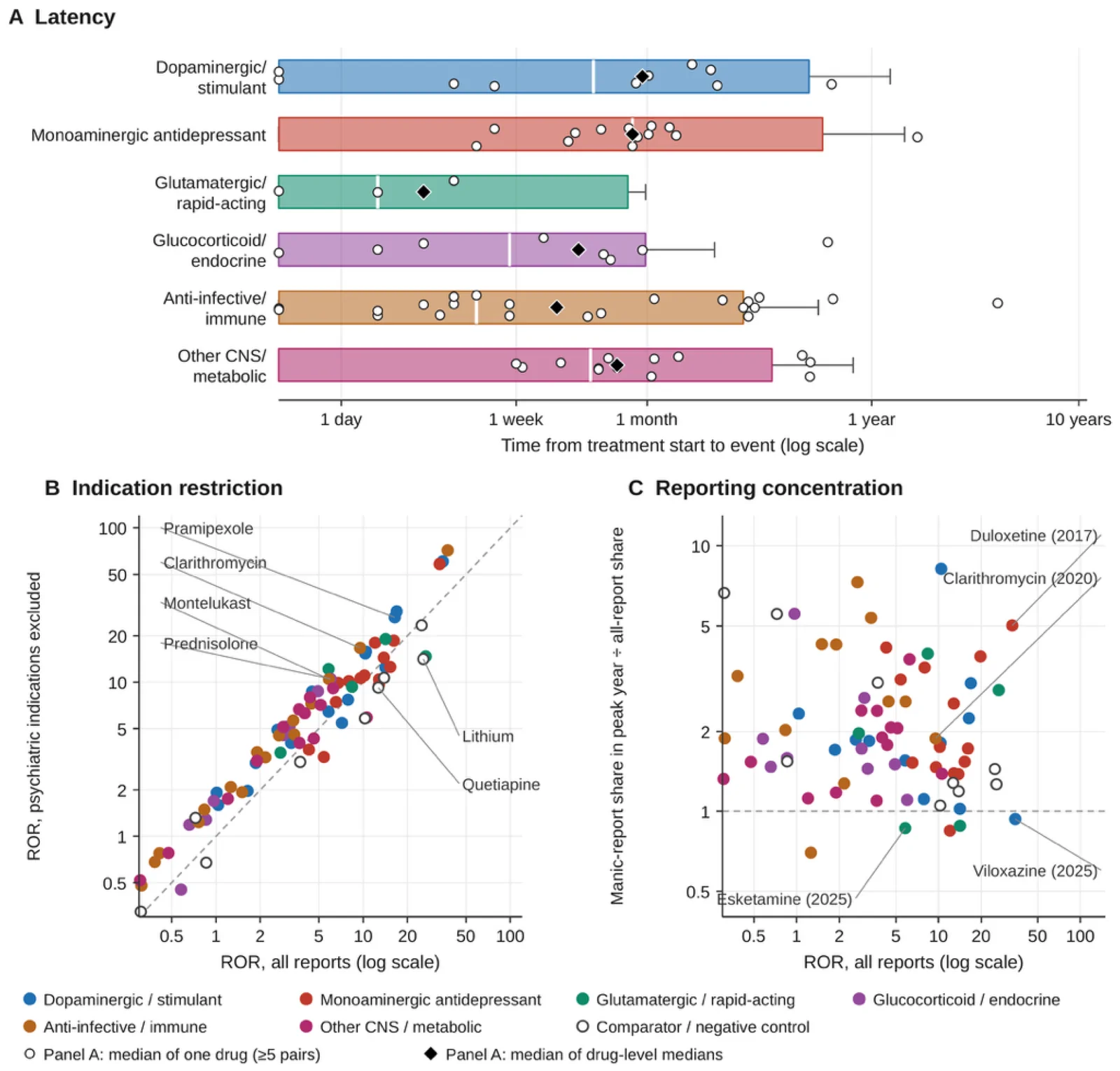
Three properties of the reported cases. (**A**) Interval from recorded treatment start to event by mechanistic group (*n* = 2074 drug–report pairs with both dates in the six groups shown; 2788 including indication comparators and negative controls; Data S1); boxes show the interquartile range of drug–report pairs and the white line the pooled median. Small open circles show the median for each drug contributing at least five pairs, and the black diamond the median of those drug-level medians, so that each drug carries equal weight (Supplementary Table S5). Latency is plotted as reported days + 0.5 so that same-day onsets can be displayed on a logarithmic axis. A sensitivity analysis restricted to reports carrying complete eight-digit start and event dates is given in Supplementary Table S2 and preserves the ordering of pooled medians. (**B**) Reporting odds ratio before and after excluding all reports carrying a psychiatric indication; points above the diagonal indicate signals that strengthen under restriction. (**C**) Temporal-concentration flag: the share of a drug’s manic reports falling in its peak calendar year, divided by the share of all of that drug’s reports falling in the same year, plotted against the reporting odds ratio. Values near 1 indicate that manic reports follow the drug’s own reporting history; values well above 1 indicate disproportionate temporal clustering. Restricted to agents with at least ten manic reports. The dashed line at 1 is a descriptive reference, not a validated threshold.

These analyses describe patterns of spontaneous reporting only. FAERS contains no exposure denominator, so none of the estimates below represents incidence or absolute or relative risk, and none can establish that a drug causes mania; they are used solely to examine whether reporting patterns are compatible with, or contrary to, the proposed framework. Three results bear on the argument of this review; these are shown in Figure 4 and Figure 5 and tabulated in full in Table 3.

Signals were distributed across pharmacologically and mechanistically distinct drug groups, whereas none of the negative controls met the signal criteria. Of the 123 ingredients examined, 104 accrued at least three manic or hypomanic reports and 66 met the signal criteria. Signals were observed among dopaminergic and stimulant agents (pramipexole ROR 16.4, 95% CI 12.3–21.8; viloxazine 34.8, 22.8–53.1), glucocorticoids (prednisolone 6.0, 5.1–7.1), glutamatergic agents (ketamine 26.6, 18.7–37.8; esketamine 5.8, 4.6–7.3), anti-infectives (clarithromycin 9.5, 7.6–11.9), and other central nervous system agents (baclofen 6.2, 5.0–7.8). None of the six negative controls met the signal criteria; their ROR point estimates ranged from 0.09 for metformin to 0.86 for omeprazole. This provides a useful internal calibration and argues against the observed pattern being a trivial consequence of overall report volume, although six negative controls cannot establish the validity or specificity of the outcome definition.

Reported latency differed across mechanistic groups in a direction broadly compatible with the proposed framework. Among the 2788 drug–report pairs for which both a treatment start date and an event date were available, the pooled median interval from initiation to event was 1 day for glutamatergic agents, 4 days for anti-infectives, 6 days for glucocorticoids, 16 days for dopaminergic and stimulant agents, and 25 days for monoaminergic antidepressants (Figure 5A). This ordering was not imposed by the analysis and broadly parallels latency patterns described in the clinical literature, including the approximately four-day onset reported across 34 published clarithromycin cases by Marković et al. [17] and the days-to-two-weeks window described for corticosteroid-associated states by De Bock and Sienaert [2]. A prespecified sensitivity analysis restricted to records with complete eight-digit treatment-start and event dates preserved the overall ordering across mechanistic groups (Supplementary Table S2).

Because pooled medians give greater weight to drugs contributing many reports, latency was also summarized at the drug level, as the median of per-drug medians among drugs contributing at least five drug–report pairs, so that each drug carried equal weight (Figure 5A; Supplementary Table S5). The contrast between the extremes was preserved: 2 days for glutamatergic agents (4 drugs) against 25 days for monoaminergic antidepressants (13 drugs). The intermediate ordering was less stable. Drug-level medians were 10.5 days for anti-infective and immune agents (22 drugs), 13.5 days for glucocorticoid and endocrine agents (8 drugs), and 28 days for dopaminergic and stimulant agents (10 drugs), so that the last group moved from fourth to fifth position. These shifts arose largely because several plotting groups combine agents from different mechanistic axes whose report volumes differ. At the level of the axes themselves, drug-level medians were 3 days for anti-infective agents (9 drugs) against 69 days for immunomodulatory agents (13 drugs), 5.5 days for glucocorticoids (6 drugs) against 126 days for testosterone and levothyroxine (2 drugs), and 5 days for psychostimulants (7 drugs) against 49 days for dopamine agonists and levodopa (3 drugs). With 4 to 22 drugs per group, drug-level estimates were imprecise, and differences across the five groups were not statistically significant (Kruskal–Wallis test on drug-level medians across these five groups, the heterogeneous other CNS/metabolic group being excluded, *p* = 0.16). The latency data therefore support a coarse separation between rapidly acting exposures, namely glutamatergic agents, anti-infective agents, and glucocorticoids, with drug-level medians of 2 to 5.5 days, and slower exposures such as antidepressants and dopamine agonists, rather than a fine-grained ordering of all groups. These findings are descriptive, and dates recorded in spontaneous reports are inherently imprecise; nevertheless, the presence of distinct reported timescales across pharmacologically different groups is compatible with the proposed framework, although it does not establish that the mechanisms themselves generated the observed ordering.

Restriction by recorded psychiatric indication shifted the disproportionality estimates differently across drug classes. This analysis assesses the dependence of the estimates on recorded indication; it cannot determine whether a particular signal is confounded, because FAERS indication fields are reporter-entered and frequently incomplete. Excluding reports carrying a recorded psychiatric indication attenuated the estimates for several psychotropic agents, consistent with a contribution from indication-related reporting (lithium 25.6 to 14.1; quetiapine 12.7 to 9.2). In contrast, the same restriction increased the point estimates for several agents used predominantly outside psychiatry (clarithromycin 9.5 to 16.7; pramipexole 16.4 to 26.3; prednisolone 6.0 to 10.5; montelukast 5.9 to 10.5; Figure 5B). These divergent changes show that restriction by recorded psychiatric indication does not affect all drug classes uniformly. Persistence or strengthening of a signal after restriction makes recorded psychiatric indication alone a less sufficient explanation for that signal, but does not remove residual confounding or establish a causal drug effect.

Two further observations concern the reporting process rather than the proposed biology. First, disproportionality estimates varied by reporter qualification. Consumers submitted 4702 out of the 11,698 mania or hypomania reports and physicians 2935; for several psychotropic agents, consumer-derived estimates exceeded the corresponding physician-derived estimates by up to about 3.6-fold (quetiapine 21.3 versus 8.5; lithium 58.6 versus 16.2), whereas for duloxetine, the physician-derived estimate was higher (91.6 versus 18.3) and for clarithromycin they were nearly identical (6.9 versus 6.8). Reporter type therefore appears to be an important source of heterogeneity in estimated disproportionality and should not be treated as incidental to interpretation.

Second, some signals showed marked temporal concentration. For duloxetine, 500 out of 802 mania or hypomania reports were submitted in a single year. Because a raw single-year proportion can also be high for a newly marketed drug or one whose overall reporting is temporally concentrated, I compared, for each drug, the proportion of manic reports occurring in its peak year with the proportion of all reports for that drug occurring in the same year. I used their ratio as a descriptive temporal-concentration flag rather than as a validated diagnostic (Figure 5C; Supplementary Table S3). Duloxetine had a ratio of 5.0, whereas viloxazine and esketamine each had a ratio of 0.9 despite relatively high raw peak-year shares. The flag cannot distinguish among publicity, litigation, batch submission, product launch, changes in market share, or other reporting dynamics and is unstable when manic report counts are small. The large ROR for duloxetine should therefore be interpreted cautiously because its manic reports are unusually concentrated relative to the drug’s own overall reporting history; the temporal pattern does not, by itself, establish whether the underlying explanation is pharmacological or reporting-related. The flag is also non-specific: among agents with at least ten manic reports, several had higher ratios than duloxetine, including ropinirole (8.2) and two negative controls (amlodipine 6.7; levocetirizine 5.5; Supplementary Table S3).

### 3.5. Susceptibility: Why the Same Exposure Produces Mania in a Minority

Across the literature reviewed in Section 3.2, manic responses occurred in only a subset of exposed individuals, but the characteristics of that subset differ substantially by drug class. These differences suggest that susceptibility is unlikely to be captured by a single construct such as “bipolar vulnerability”. In adults with bipolar disorder initiating methylphenidate, Viktorin et al. [10] found an increased manic hazard during monotherapy but no excess hazard among those receiving a concurrent mood stabilizer. This finding suggests effect modification by treatment context within bipolar disorder, although replication has been inconsistent (Section 3.2.2); it does not establish that a bipolar diathesis is required, because mania-specific risk in adults without bipolar disorder remains largely unquantified. At the other end of the spectrum, 93.2% of patients in the TNF-α inhibitor case series had no reported psychiatric history, although absence of a documented history cannot exclude latent susceptibility. In the randomized testosterone study, 8 out of 50 men receiving the full supraphysiologic dose developed mild or marked hypomanic symptoms, and responders could not be distinguished from nonresponders by the baseline demographic, psychological, laboratory, or physiological measures collected [56]. Together, these observations argue for multiple forms of susceptibility rather than a single cross-class diathesis.

Four candidate susceptibility domains are useful to distinguish. The first is clinical. A personal or family history of bipolar disorder, previous treatment-emergent switching, mixed features, and early-onset or highly recurrent depression are plausible markers of vulnerability, particularly in the literature derived from mood-disorder populations. Their predictive value, however, is not established uniformly across pharmacological classes. For corticosteroids, dose-related neuropsychiatric risk is comparatively well-documented, but this does not show that clinical host factors are irrelevant or that dose overrides them. The relative contribution of drug exposure and baseline clinical vulnerability is therefore likely to differ by class rather than follow a single hierarchy.

The second domain is pharmacokinetic and is attractive because exposure is, at least in principle, directly measurable. Where a mania-associated drug is metabolized by a polymorphic enzyme, or is co-administered with an inhibitor or inducer affecting its disposition, apparently idiosyncratic responses may partly reflect differences in effective exposure (Section 3.3.7). Joas et al. [181] reported an association between CYP2C19 polymorphisms, antidepressant prescribing patterns, and treatment-emergent mania in bipolar disorder. Clarithromycin provides a clinically plausible interaction setting because it inhibits CYP3A4, and several published cases of clarithromycin-associated mania involved concomitant corticosteroid treatment or other potentially interacting drugs. Such observations support pharmacokinetic interaction as a hypothesis, but they do not establish it as the cause of those episodes. Direct concentration data remain notably absent from the published drug-induced mania literature.

The third domain is pharmacodynamic and genomic. Genome-wide studies of antidepressant-associated mania have been undertaken [182], meta-analytic work has sought to identify clinical risk markers for it [183], and pharmacogenomic overlap between antidepressant treatment response and antidepressant-associated treatment-emergent mania has been reported [180]. These findings remain exploratory and have not yielded clinically validated predictors. Candidate-gene associations involving dopaminergic, circadian, and related pathways likewise remain insufficiently replicated for clinical prediction. Genomic susceptibility is therefore biologically plausible but currently more useful for hypothesis generation than for individual risk stratification.

The fourth domain is chronobiological. Section 3.3.3 identifies sleep and circadian disruption as a candidate mediator through which several pharmacologically distinct exposures might contribute to manic activation, but mediation has not been demonstrated. No study identified in my search formally modeled the sequence from drug exposure to objective sleep change to subsequent manic switching. If such mediation occurs, premorbid features such as irregular sleep–wake timing, evening chronotype or a history of mood destabilization after sleep loss might identify a susceptible subgroup across otherwise unrelated drug classes. This hypothesis is directly testable using prospective actigraphy or other longitudinal sleep monitoring beginning before or at treatment initiation.

Age and sex may further modify observed susceptibility, but the available evidence is largely descriptive. Approximately two-thirds of published antibiomania cases were male [7], but case-series composition cannot establish sex-specific risk in the absence of an exposure denominator. In my post-marketing dataset, age information was too incomplete for stratified estimates to support more than descriptive interpretation. These demographic variables should therefore be treated as candidate modifiers rather than established predictors.

## 4. Discussion

### 4.1. What the Post-Marketing Analysis Cannot Establish

The limitations are central to interpretation. Spontaneous reports provide no exposure denominator, so no estimate presented here represents incidence or absolute clinical risk. The ROR instead reflects the relative composition of a drug’s report stream and is therefore sensitive to the other events reported for that drug. A particularly discordant example is provided by the TNF-α inhibitors: despite published case-level evidence of manic states, including cases without a recorded psychiatric history [8], adalimumab and etanercept showed RORs well below unity (adalimumab 0.15, 95% CI 0.12–0.20; etanercept 0.05, 0.03–0.09). These inverse estimates should not be interpreted as evidence of protection or as refuting the case literature. Both agents generate very large report streams dominated by other adverse-event categories, creating conditions in which a rare psychiatric event may be masked by the composition of the report stream. Masking is therefore a plausible explanation for the inverse estimates, but it cannot be established from the present analysis alone. More generally, neither an elevated nor an inverse ROR should be interpreted symmetrically as evidence for causation or absence of risk.

Three additional limitations are important. First, the MedDRA preferred terms Mania and Hypomania depend on reporter recognition and coding practice and do not capture the full range of subsyndromal activation discussed in Section 3.1; milder phenotypes are therefore likely to be incompletely represented. Second, designation of a drug as a “primary suspect” reflects the reporter’s causal attribution and may itself be influenced by existing knowledge or expectations about the drug. Third, low or absent reporting cannot distinguish a genuinely rare event from limited exposure, low reporting propensity or failure to recognize the association. Immune checkpoint inhibitors illustrate this problem: few manic or hypomanic reports were identified for these agents (3 out of 59,688 reports for pembrolizumab, 9 out of 68,212 for nivolumab, and 1 out of 15,190 for ipilimumab; Supplementary Table S1), counts that cannot distinguish among these possibilities. Mania and hypomania were also not analyzed as separate outcomes in the principal pharmacovigilance study of checkpoint inhibitors [104]. Conversely, manic states have been reported after chimeric antigen receptor T-cell therapy [184], indicating that the paucity of checkpoint-inhibitor reports should not be generalized to immunotherapeutic interventions as a whole.

### 4.2. Using the Mismatches Between Mechanism and Data

The most informative use of this analysis is not the catalogue of reporting signals itself, but the discrepancies between mechanistic expectation and observed reporting patterns. Levodopa, despite its direct relevance to the dopaminergic mechanisms discussed in Section 3.3.1, produced the weakest signal among the dopaminergic agents meeting the signal criteria (ROR 1.9, 95% CI 1.5–2.3). One possible explanation is phenotypic and coding mismatch: behavioral activation associated with dopaminergic therapy in Parkinson’s disease is often characterized as impulse-control disorder or dopamine dysregulation syndrome rather than as mania. The present analysis cannot determine whether this accounts for the relatively weak levodopa signal. Conversely, viloxazine and zuranolone produced comparatively large RORs despite small numbers of manic or hypomanic reports and little or no corresponding case literature. These findings are therefore hypothesis-generating rather than evidence of clinical risk and are better suited to evaluation in denominator-based cohort studies. Each of these mismatches generates a specific, testable question; Table 4 summarizes those for which an appropriate study design can be specified.

### 4.3. Clinical Implications and a Research Agenda

The clinical implications of the evidence reviewed here are necessarily limited by the absence of denominator-based risk estimates for many drug classes. The post-marketing analysis in Section 3.4 cannot provide absolute risk or define evidence-based monitoring intervals, but the reported latency patterns, considered together with the clinical literature, suggest that the timing of vigilance may reasonably differ across exposures. Antibiotic-associated cases and many corticosteroid-associated reactions have been reported within days to the first few weeks of treatment, whereas stimulant-associated mania in bipolar disorder is quantified over the first several months, and behavioral complications of dopaminergic therapy may emerge after continued exposure or dose escalation. Ketamine- and esketamine-associated switching is less readily assigned to a single early window because events have also been reported during maintenance treatment. These patterns support attention to temporal context rather than a uniform surveillance schedule.

Three practical implications can be stated more cautiously. First, a reduced need for sleep—particularly when accompanied by increased energy or behavioral activation—is clinically relevant because it is a characteristic feature of mania and may precede or accompany mood destabilization. Whether sleep disturbance mediates pharmacologically induced switching, however, has not been demonstrated, so sleep monitoring should be regarded as a pragmatic clinical observation rather than a validated predictive marker. Second, concurrent mood-stabilizing treatment was associated with a substantially lower manic hazard during methylphenidate treatment in patients with bipolar disorder in one Swedish registry study [10], a pattern that has not been consistently replicated (Section 3.2.2), and this finding should not be generalized to other pharmacological triggers without supporting evidence. A case series reporting rapid improvement of corticosteroid-induced mania with sodium valproate [71] suggests that an established episode may sometimes be managed pharmacologically when corticosteroid treatment remains medically necessary, but does not establish a general treatment strategy. Third, improvement after dose reduction or withdrawal can strengthen causal attribution in an individual case, particularly when the temporal relationship is close, but dechallenge is neither specific nor sufficient because spontaneous remission and concurrent treatment changes may contribute. Decisions to reduce or discontinue an implicated drug must therefore depend on the severity of the manic state, the necessity of the underlying treatment, and the availability of alternatives.

The research agenda follows directly from the gaps identified in this review. Five priorities are especially tractable. (i) Denominator-based estimates. For antibiotics, TNF-α inhibitors and interferons, I identified little or no mania-specific denominator-based evidence. Linked prescribing, clinical, and hospital datasets could support active-comparator cohort studies or other population-based designs to estimate absolute and relative risk, while self-controlled designs could assess within-person changes in incidence around exposure. (ii) A formal test of sleep as a mediator. Prospective measurement of sleep and circadian timing before and after drug initiation, using actigraphy or validated wearable measures together with repeated mood assessment, could determine whether sleep disturbance mediates switching or occurs in parallel with it. (iii) Prospective study of clinically indicated high-dose corticosteroid exposure. Rather than experimentally inducing exposure, patients already receiving high-dose or pulse corticosteroids for oncological, rheumatological, or other medical indications could undergo repeated standardized assessments of activation, sleep, and mood. This would provide contemporary exposure–response data without requiring deliberate induction of a potentially serious psychiatric adverse effect. (iv) Phenotype harmonization. Mania, hypomania, subsyndromal activation, dopamine dysregulation syndrome, and impulse-control disorders are currently captured through partially separate clinical and coding frameworks. Harmonized prospective phenotyping would test whether the relatively weak levodopa mania signal identified in Section 4.2 reflects true biological differences or differences in ascertainment and terminology. (v) Direct testing of network convergence. Functional imaging obtained during pharmacologically associated manic or activation states and repeated after remission could be compared with the lesion-derived network identified by Cotovio et al. [22], providing a direct test of the inverse-mapping framework proposed in Section 3.3.6.

### 4.4. Status of the Convergence Hypothesis

The framework developed here should be read as a hypothesis requiring validation rather than as an established mechanism, and its components rest on evidence of different kinds. That several drug classes can precipitate manic states is supported, for some classes, by randomized, cohort, or self-controlled evidence (Section 3.2). That each class acts principally through the proximal mechanism assigned to it in Figure 2 is an inference from the pharmacology and from experimental and clinical studies of each mechanism in isolation (Section 3.3); no study has measured the proposed mechanism in patients developing drug-induced mania. That the five mechanisms converge on the ventral prefrontal–amygdala–striatal network is extrapolated from the lesion and imaging literature on secondary and idiopathic mania (Section 3.3.6) and has not been examined in pharmacologically induced states. The post-marketing analysis does not close these gaps: it describes reporting patterns without exposure denominators, cannot establish that any drug causes mania, and shows a latency pattern that is compatible with, but not specific to, the proposed mechanisms. The hypothesis would be weakened if prospective studies found no consistent relation between the mechanism engaged and the timing or phenotype of activation, if sleep change neither preceded nor mediated switching across drug classes, or if functional imaging during pharmacologically associated activation showed no involvement of the lesion-derived network.

## 5. Conclusions

More than three decades after the cross-class synthesis of Peet and Peters [11], the literature on drug-induced mania remains fragmented across pharmacological classes, with no integrated account of why mechanistically diverse exposures can be associated with a similar clinical state. This review proposes that such episodes may arise through a limited number of proximal mechanisms—involving dopaminergic gain and incentive sensitization, glutamatergic plasticity, sleep and circadian disruption, glucocorticoid signaling, and neuroimmune signaling—whose effects may converge on circuitry overlapping the lesion-defined network implicated in secondary mania. The specific mappings between individual drug classes, these mechanisms, and the convergence network remain unevenly supported, and in several instances, explicitly hypothetical. The convergence model is therefore a hypothesis requiring validation: neither the assignment of drug classes to proximal mechanisms nor the convergence of those mechanisms on the lesion-defined network has been tested directly.

Viewed through this framework, pharmacological exposures can be used as reversible, and in some settings, dose-graded probes of the systems capable of destabilizing mood rather than considered only as isolated adverse reactions. The exploratory cross-class reporting analysis is compatible with this approach but cannot test it, because spontaneous reports lack exposure denominators and cannot establish causality: reported latency separated rapidly acting from slower pharmacologically distinct groups in a direction broadly compatible with the proposed framework, although a finer ordering of groups was not robust to summarizing latency at the drug level, while restriction by recorded psychiatric indication affected drug classes differently. These patterns do not establish the mechanisms proposed here, and the same analysis illustrates the substantial influence of indication, reporter behavior, coding, and report-stream composition on spontaneous-report estimates.

The principal value of a cross-class approach is therefore not to assign a single mechanism to drug-induced mania, but to generate testable comparisons across pharmacologically distinct exposures. Prospective measurement of exposure, sleep and circadian change, standardized manic phenotypes, denominator-based risk estimates, and direct imaging of pharmacologically associated activation against the lesion-derived network could now test the links that remain inferential. Treating drug-associated manic states as structured pharmacological perturbations may provide a route from a fragmented adverse-event literature toward a testable neurobiology of manic activation.

## Figures and Tables

**Table 1:** Competing designations for pharmacologically precipitated manic states.

Term	Source	What It Requires	What It Excludes	Consequence for Case Ascertainment
Secondary mania	Krauthammer and Klerman [20]	A manic syndrome with an identified antecedent physical illness or drug	Mania in established bipolar disorder	Groups pharmacological and structural causes; the framework this review inherits
Substance/medication-induced bipolar and related disorder	DSM-5	Onset during intoxication, withdrawal or exposure; not better explained by an independent bipolar disorder	Episodes in patients with a pre-existing bipolar diagnosis	Excludes precisely the population in which switching is most often studied
Treatment-emergent affective switch (TEAS)	Bipolar treatment literature	Emergence of manic or hypomanic symptoms during treatment of a depressive episode	Activation arising outside a mood-disorder indication	Restricts the phenomenon to psychiatric care
Treatment-emergent mania	Register and cohort studies	A recorded manic episode or diagnosis after drug initiation	Subsyndromal activation not reaching diagnostic threshold	Ascertains only hospital- or diagnosis-level events
Antibiomania	Antibiotic case literature	Manic symptoms during antibacterial therapy, with or without bipolar diathesis	Nothing systematically	Maximally inclusive; no denominator has ever been established

DSM-5, Diagnostic and Statistical Manual of Mental Disorders, fifth edition; TEAS, treatment-emergent affective switch.

**Table 2:** Drug classes precipitating manic states, ordered by the strongest available study design.

Drug Class	Strongest Design Available	Best Available Estimate	Typical Latency	Reversible on Withdrawal	Bipolar Diathesis Required
Anabolic-androgenic steroids	Randomized placebo-controlled crossover	16% hypomanic at 600 mg/week testosterone (12% mild, 4% marked; *n* = 50)	Weeks of supraphysiologic dosing	Yes (crossover design)	No; and no baseline predictor identified
Antidepressants (bipolar depression)	Network meta-analysis of RCTs; target trial emulation	No agent significantly above placebo; naturalistic estimates 17.3–48.8%	Weeks	Usually	Yes for the highest rates
Corticosteroids	Population cohort with same-disease unexposed comparators; meta-analysis	Mania HR 4.35 (3.67–5.16); manic more frequent than depressive symptoms acutely, OR 2.42 (1.48–3.97)	1–2 weeks; dose threshold above 40 mg/day prednisone-equivalent	Usually rapid on taper	No
Psychostimulants	Meta-analysis of RCTs (null); national register, within-individual	HR 6.7 (2.0–22.4) on monotherapy; HR 0.6 (0.4–0.9) with a mood stabilizer	First 3 months	Generally	Studied almost only in bipolar populations
Dopaminergic antiparkinsonian agents	Experimental levodopa challenge (hypomania); large cross-sectional study (impulse-control disorders)	Impulse control disorders 13.6% of 3090 patients; agonist versus no agonist OR 2.72 (2.08–3.54)	Months to years; dose-cumulative	Usually on dose reduction	No
Ketamine and esketamine	Propensity-matched cohort; clinical cohort	No increase under mood-stabilizer cover; 28.9% during maintenance in one uncontrolled service cohort	Days (acute) to months (maintenance)	Generally self-limited	Not strictly
Classic psychedelics	Systematic review with meta-analysis	5.8% in supervised trials; 30% in naturalistic bipolar samples	Acute, within session	Typically acute and self-limited	Strongly modifying, not required
Interferons	Prospective uncontrolled cohort	Mania in 3 and irritable hypomania in 15 out of 93 treated patients	Weeks to months	Reported	Unclear
Antibiotics (macrolides, quinolones, antimycobacterials)	Systematic reviews of case material	No denominator exists; 47 and 34 pooled cases	About 4 days (clarithromycin)	Essentially always; rechallenge positive in 5 of 5 tested	No
Tumor necrosis factor-α inhibitors	Systematic review incorporating one cohort	40 out of 7600 patients (0.53%) with elated mood	Early for infliximab; later for adalimumab and etanercept	Reported	No; 93.2% had no psychiatric history
Immune checkpoint inhibitors	None	Not estimable; no case report, case series, cohort or systematic review identified	Unknown	Unknown	Unknown
Other central nervous system agents (levetiracetam)	Systematic review of case reports	Not estimable; causality frequently doubtful on formal appraisal	Variable	Usually	Frequently present
Withdrawal and discontinuation states	Self-controlled case series (null); case reports	Glucocorticoid discontinuation showed no significant mania signal (*n* = 21,995)	Days after cessation	Usually	Unclear

Estimates are the best available and are not comparable across rows. Latency and reversibility are as reported in the sources cited in Section 3.2. HR, hazard ratio; OR, odds ratio; RCT, randomized controlled trial.

**Table 3:** Reporting odds ratios for mania or hypomania in the FDA Adverse Event Reporting System, 2015Q1–2026Q1, for selected agents grouped by mechanistic group.

Agent	Mania/ Hypomania Reports	Total Reports	ROR (95% CI)	IC025	ROR Excluding Psychiatric Indications	Median Latency, Days
**Dopaminergic/stimulant**						
Viloxazine	22	857	34.77 (22.76–53.11)	3.58	60.65	30 (*n* = 13)
Cabergoline	19	1502	16.90 (10.74–26.58)	2.80	28.78	–
Pramipexole	48	3923	16.38 (12.31–21.78)	3.32	26.35	49 (*n* = 11)
Atomoxetine	71	6714	14.16 (11.20–17.90)	3.28	12.46	5 (*n* = 20)
Methylphenidate	124	28,449	5.82 (4.87–6.95)	2.20	6.44	0 (*n* = 56)
Lisdexamfetamine	90	15,220	7.89 (6.41–9.71)	2.56	7.69	61 (*n* = 30)
Levodopa	98	69,663	1.86 (1.53–2.27)	0.55	2.99	26 (*n* = 45)
**Monoaminergic antidepressant**						
Duloxetine	802	35,007	33.08 (30.77–35.57)	4.77	58.33	30 (*n* = 119)
Escitalopram	194	16,282	16.13 (13.99–18.61)	3.68	18.59	41 (*n* = 73)
Sertraline	387	38,445	13.82 (12.48–15.30)	3.54	14.42	4 (*n* = 140)
Venlafaxine	258	27,285	12.83 (11.34–14.53)	3.40	10.42	31 (*n* = 102)
Bupropion	158	32,363	6.54 (5.58–7.65)	2.40	7.44	5 (*n* = 42)
Amitriptyline	13	4018	4.28 (2.48–7.38)	0.99	3.64	18 (*n* = 10)
**Glutamatergic/rapid-acting**						
Ketamine	32	1621	26.59 (18.73–37.76)	3.64	14.68	1 (*n* = 9)
Zuranolone	14	1315	14.19 (8.38–24.03)	2.37	19.04	0 (*n* = 7)
Memantine	27	4273	8.39 (5.75–12.26)	2.23	9.33	–
Esketamine	74	16,906	5.82 (4.63–7.32)	2.10	12.16	3 (*n* = 25)
Dextromethorphan	47	22,692	2.74 (2.06–3.65)	0.94	3.48	3 (*n* = 9)
**Glucocorticoid/endocrine**						
Prednisolone	146	32,461	6.01 (5.11–7.08)	2.27	10.50	2 (*n* = 75)
Methylprednisolone	78	21,037	4.93 (3.94–6.16)	1.88	8.73	1 (*n* = 39)
Prednisone	90	37,734	3.17 (2.57–3.90)	1.29	5.19	9 (*n* = 61)
Dexamethasone	94	43,442	2.87 (2.34–3.52)	1.16	4.53	20 (*n* = 42)
Testosterone	23	31,379	0.97 (0.64–1.46)	−0.75	1.70	224 (*n* = 12)
**Anti-infective/immune**						
Chloroquine	9	325	37.54 (19.35–72.84)	2.53	71.37	–
Clarithromycin	79	11,063	9.53 (7.63–11.90)	2.79	16.68	1 (*n* = 29)
Montelukast	64	14,523	5.86 (4.58–7.49)	2.07	10.46	3 (*n* = 47)
Isoniazid	7	2719	3.40 (1.62–7.14)	0.25	4.57	87 (*n* = 5)
Ciprofloxacin	48	29,334	2.16 (1.63–2.87)	0.61	3.25	4 (*n* = 19)
Adalimumab	54	451,372	0.15 (0.12–0.20)	−3.11	0.27	69 (*n* = 30)
Etanercept	10	283,328	0.05 (0.03–0.09)	−5.44	0.08	15 (*n* = 6)
Interferon beta	12	79,340	0.20 (0.11–0.35)	−3.26	0.37	104 (*n* = 7)
Isotretinoin	22	34,801	0.83 (0.55–1.27)	−0.97	1.49	92 (*n* = 7)
**Other CNS/metabolic**						
Lamotrigine	226	28,885	10.57 (9.26–12.06)	3.12	5.90	7 (*n* = 79)
Baclofen	79	16,830	6.25 (5.00–7.80)	2.21	9.13	184 (*n* = 15)
Topiramate	79	20,443	5.14 (4.12–6.41)	1.94	7.12	11 (*n* = 49)
Levetiracetam	155	47,576	4.35 (3.71–5.10)	1.82	7.96	21 (*n* = 36)
Zolpidem	52	14,933	4.62 (3.52–6.07)	1.69	4.31	19 (*n* = 27)
Semaglutide	22	60,839	0.47 (0.31–0.72)	−1.77	0.78	17 (*n* = 9)
**Indication comparator**						
Lithium	78	4110	25.64 (20.48–32.11)	4.06	14.13	56 (*n* = 26)
Aripiprazole	882	51,050	24.96 (23.29–26.75)	4.38	23.38	4 (*n* = 316)
Quetiapine	363	39,209	12.67 (11.41–14.07)	3.41	9.21	23 (*n* = 128)
Valproate	147	19,191	10.28 (8.73–12.11)	3.02	5.82	38 (*n* = 48)
**Negative control**						
Omeprazole	20	30,751	0.86 (0.55–1.33)	−0.97	0.67	31 (*n* = 23)
Levocetirizine	22	39,960	0.72 (0.48–1.10)	−1.17	1.32	4 (*n* = 18)
Amlodipine	11	46,920	0.31 (0.17–0.56)	−2.67	0.32	–
Losartan	3	15,283	0.26 (0.08–0.80)	−3.86	0.49	–
Metformin	5	73,310	0.09 (0.04–0.21)	−4.91	0.14	–
Atorvastatin	2	52,099	–	–	–	–

Based on 15,419,124 deduplicated cases, of which 11,698 carried a preferred term of Mania or Hypomania. Estimates are for the drug recorded as the primary suspect. Median latency is computed from drug–report pairs in which the drug was recorded as a primary or secondary suspect, and is shown only where at least five pairs carried both a treatment start date and an event date. Reporting odds ratios describe disproportionality within spontaneous reports and are not measures of incidence or risk. The complete table for all 123 ingredients, together with all sensitivity analyses, is given in Supplementary Table S1. Mechanistic group names are shown in bold. CI, confidence interval; IC025, lower bound of the 95% credibility interval of the information component; ROR, reporting odds ratio.

**Table 4:** Tractable questions arising from the mismatches between mechanism and observed data.

Question	Why It Is Unresolved	Design That Would Answer It
What is the incidence of mania during macrolide, antimycobacterial, tumor necrosis factor-α inhibitor and interferon therapy?	Denominator-based evidence is limited to one small prospective interferon cohort and one uncontrolled TNF-α-inhibitor cohort, and is indirect for antibiotics	Self-controlled case series in linked prescribing and hospital data, as already carried out for corticosteroids
Is pharmacologically precipitated switching mediated by sleep loss, or does it occur in parallel with it?	No study has measured sleep between exposure and switch	Wearable actigraphy from initiation, with formal mediation analysis, in patients starting corticosteroids, stimulants or dopamine agonists
Does high-dose corticosteroid exposure induce measurable activation in unselected patients?	The dose–response data are more than 50 years old and the modern literature is observational	Prospective monitored cohort with repeated activation ratings during oncological or rheumatological pulse therapy
Why does levodopa produce the weakest reporting signal of any dopaminergic agent meeting the signal criteria?	Activation in Parkinson’s disease is coded as impulse control disorder or dopamine dysregulation syndrome, not as mania	Harmonized activation phenotype applied across neurology and psychiatry coding
Do recently licensed agents with large reporting signals and no case literature (for example, viloxazine, zuranolone) carry real risk?	Signals rest on small numbers and short marketing histories	Active-comparator cohort studies in claims data
Does chronobiological instability predict switching irrespective of mechanistic axis?	Never tested	Prospective cohort with baseline chronotype and sleep regularity in patients starting a high-risk agent
Do pharmacologically induced activation states engage the lesion-derived mania network?	The inverse mapping proposed here is interpretive, not tested	Functional imaging during induced activation, compared against the lesion network of Cotovio et al. [22]

## Data Availability

The quarterly public release files of the United States Food and Drug Administration Adverse Event Reporting System analyzed in Section 3.4 are publicly available from the agency’s distribution site and are not redistributed here. All code required to reproduce the analysis, together with the complete result table for all 123 ingredients across the primary and principal sensitivity analyses, the de-identified drug–interval pairs underlying Figure 5A, and the temporal-concentration results, are provided as Supplementary Materials. No other new data were created.

## Supplementary Materials

The following supporting information can be downloaded at: https://www.mdpi.com/article/10.3390/brainsci16101081/s1. Supplementary Methods S1: Full description of the data source, ingredient mapping dictionary, deduplication procedure, disproportionality computation, and all sensitivity analyses; Table S1: Disproportionality results for all 123 ingredients across the primary and principal sensitivity analyses; Table S2: Time-to-onset analysis restricted to reports carrying complete eight-digit start and event dates; Table S3: Definition and computation of the temporal-concentration flag; Table S4: Study-design-specific evidence grades underlying Figure 3, with the sources for each cell; Table S5: Pooled and drug-level summaries of reported latency by mechanistic group and by mechanistic axis; Table S6: Search sources, search terms, and eligibility criteria; Table S7: Structured appraisal of the controlled, cohort, self-controlled and pooled studies informing strong or moderate grades; Data S1: De-identified drug–interval pairs underlying Figure 5A; Code S1: The complete analysis code, comprising the per-quarter parser, the ingredient mapping dictionary with every regular expression, the deduplication and disproportionality routines, and the code generating every figure and table.

## Abbreviations

The following abbreviations are used in this manuscript:

Abbreviation	Definition
5-HT2A	Serotonin 2A
ACTH	Adrenocorticotropic hormone
AMPA	α-Amino-3-hydroxy-5-methyl-4-isoxazolepropionic acid
BDNF	Brain-derived neurotrophic factor
CI	Confidence interval
CNS	Central nervous system
CRP	C-reactive protein
CSF	Cerebrospinal fluid
DSM-5	Diagnostic and Statistical Manual of Mental Disorders, fifth edition
FAERS	United States Food and Drug Administration Adverse Event Reporting System
FDA	United States Food and Drug Administration
GR	Glucocorticoid receptor
HPA	Hypothalamic–pituitary–adrenal
HR	Hazard ratio
IC	Information component
IC025	Lower bound of the 95% credibility interval of the information component
ICD-11	International Classification of Diseases, eleventh revision
IL	Interleukin
MedDRA	Medical Dictionary for Regulatory Activities
MR	Mineralocorticoid receptor
NMDA	N-methyl-D-aspartate
OR	Odds ratio
RCT	Randomized controlled trial
REM	Rapid eye movement
ROR	Reporting odds ratio
TEAS	Treatment-emergent affective switch
TNF-α	Tumor necrosis factor-α
YMRS	Young Mania Rating Scale
