# Supplementary material for: From Pharmacological Trigger to Manic State: Convergent Neurobiological Mechanisms of Drug-Induced Mania
Source: Brain Sci. 2026 Oct 8;16(10):1081. doi: 10.3390/brainsci16101081 (PMC13702241; doi:10.3390/brainsci16101081)
Supplement: Supplementary file 1 [file brainsci-16-01081-s001.zip › Table S7.pdf]

Supplementary Material

From pharmacological trigger to manic state: convergent neurobiological mechanisms of drug-induced mania

Ji Hyun Park

College of Pharmacy, Duksung Women's University, Seoul, Republic of Korea

Table S7: Structured appraisal of the controlled, cohort, self-controlled and pooled studies informing strong or moderate grades

Reference numbers refer to the reference list of the main text. In place of a single risk-of-bias instrument, each study is characterized by design, population, manic outcome ascertainment and principal threats to validity.

Class	Study	Design	Population	Manic outcome	Principal threats to validity
Corticosteroids	Fardet et al. [9]	Population cohort with same-disease unexposed comparators (primary care)	786,868 courses in 372,696 patients	Incident severe neuropsychiatric events, including mania, from records	Observational; residual confounding by indication and disease severity possible
Corticosteroids	Kusudo et al. [70]	Systematic review and meta-analysis	73 studies; N = 3,759,659	Manic and depressive symptoms	Heterogeneous designs; manic symptoms rather than diagnosed mania
Anabolic-androgenic steroids	Pope et al. [56]	Randomized placebo-controlled crossover	56 healthy men (50 at full dose)	YMRS; mild or marked hypomanic responses	Supraphysiologic dosing in volunteers; responders a minority (8 of 50)
Anabolic-androgenic steroids	Su et al. [59]	Double-blind, fixed-order, placebo-controlled crossover (methyltestosterone)	20 healthy men	Mood and behavioral ratings; one manic and one hypomanic response	Non-randomized order; small sample; short exposure
Anabolic-androgenic	Yates et al.	Randomized double-blind comparison	50 healthy men	Mood and behavioral measures; one	Single event, in a non-completer; small sample

steroids	[60]	of three testosterone doses		manic-like syndrome at the highest dose	
Anabolic-androgenic steroids	Tricker et al. [61]	Randomized placebo-controlled trial (600 mg/week)	43 healthy men	Anger and mood inventories	Not designed to detect mania; small sample
Anabolic-androgenic steroids	Bhasin et al. [62]	Randomized placebo-controlled trial (replacement dose)	5,204 men aged 45–80 years with hypogonadism and cardiovascular disease or increased cardiovascular risk (TRAVERSE)	Depressive symptoms (PHQ-9), mood and energy; mania not assessed or reported	Replacement rather than supraphysiologic dosing; mania not a specified outcome
Anabolic-androgenic steroids	Pope and Katz [63]	Controlled cross-sectional comparison	88 steroid users and 68 nonusers	Structured clinical interview for mood syndromes during and outside steroid use	Retrospective ascertainment; self-selected users; concomitant substance use not excluded by design
Dopaminergic antiparkinsonian agents	Murphy et al. [66]	Experimental levodopa challenge	Patients with bipolar disorder	Hypomania	Early study; small sample; not replicated with modern instruments
Psychostimulants	Chiorean et al. [4]	Systematic review and meta-analysis of randomized studies	5 studies; n = 1653	Manic symptom scores	Mostly stabilized patients on mood stabilizers; little information on monotherapy
Psychostimulants	Viktorin et al. [10]	National register, within-individual design	2,307 adults with bipolar disorder	Mania within three months of methylphenidate initiation	Controls stable confounders but not time-varying confounding; bipolar population only
Psychostimulants	Jefsen et al. [72]	National register, mirror-image design	1,043 patients with bipolar disorder	Manic episodes one year before and after initiation	Mirror-image design sensitive to regression from a pre-treatment peak; bipolar population only
Psychostimulants	Vidal et al. [73]	Nationwide claims database, self-controlled analyses	Adults with bipolar disorder (2,745 without and 3,526 with mood stabilizers)	Inpatient mania diagnoses	Excess without mood stabilizers disappeared in case-crossover analyses; natural course of illness a competing explanation
Antidepressants	Oliva et al. [3]	Network meta-analysis of randomized trials	13 trials; 1362 patients with bipolar depression	Treatment-emergent switch	Stabilized patients and short follow-up; wide confidence intervals
Antidepressants	Rohde et al. [67]	Target trial emulation in national registers	979 patients with bipolar depression	Mania over one year	Observational; residual confounding possible
Antidepressants	Barbuti et	Systematic review of naturalistic	32 studies	Treatment-emergent mania or	Cannot separate treatment-emergent switching

	al. [68]	studies		hypomania	from the natural cyclicity of bipolar disorder
Ketamine / esketamine	Liu et al. [77]	Propensity-matched cohort	2126 patients with bipolar depression	Manic switch at 1–180 days	Confounding by indication and treatment selection; mood-stabilizer co-treatment throughout
Ketamine / esketamine	Santucci et al. [78]	Uncontrolled clinical service cohort	45 patients; 518 patient-months	Manic or hypomanic episodes during induction and maintenance	No comparator; cannot separate exposure from illness course
Classic psychedelics	Eskinazi et al. [19]	Systematic review and meta-analysis	23 studies (4 in meta-analysis)	Hypomanic or manic symptoms; transition to bipolar disorder	Heterogeneous designs; naturalistic samples differ in population and setting from supervised trials
Interferons	Constant et al. [38]	Prospective uncontrolled cohort	93 interferon-naive patients	Systematically assessed manic, hypomanic and mixed states	No comparator; small number of frank manic episodes (3)
Montelukast	Paljarvi et al. [88]	Propensity-matched cohort	154,946 patients	Prespecified incident manic episode or bipolar disorder among other diagnoses	Observational; single study; null result
Montelukast	Wintzell et al. [87]	National active-comparator cohort	74,291 children	Psychiatric composite that did not include mania	Outcome does not isolate mania (contributes to the moderate grade only as context)
Withdrawal states	Fardet et al. [106]	Self-controlled case series	21,995 patients discontinuing long-term glucocorticoids	Prespecified mania, depression and delirium	Restricted to glucocorticoid discontinuation; null for mania
