# Supplementary material for: From Pharmacological Trigger to Manic State: Convergent Neurobiological Mechanisms of Drug-Induced Mania
Source: Brain Sci. 2026 Oct 8;16(10):1081. doi: 10.3390/brainsci16101081 (PMC13702241; doi:10.3390/brainsci16101081)
Supplement: Supplementary file 1 [file brainsci-16-01081-s001.zip › Table S6.pdf]

Supplementary Material

From pharmacological trigger to manic state: convergent neurobiological mechanisms of drug-induced mania

Ji Hyun Park

College of Pharmacy, Duksung Women's University, Seoul, Republic of Korea

Table S6: Search sources, search terms, and eligibility criteria

For the literature synthesis described in Section 2.1 and Figure 1A of the main text.

Item	Description
Sources	Europe PMC REST interface (indexing MEDLINE/PubMed, PubMed Central and preprint servers); targeted web searching; backward citation-chasing; hand-searching of the reference lists of every included review
Date of final search	2 September 2026 (Final)
Limits	No language restriction and no lower date limit; additional searches with French, Spanish and German terms
Concept block 1: outcome (title/abstract)	mania; manic; hypomania; hypomanic; manic switch; secondary mania; antibiomania; bipolar disorder
Concept block 2: exposure	Individual agents and drug classes treated in Section 3.2: corticosteroids/glucocorticoids; anabolic-androgenic steroids and testosterone; antidepressants; psychostimulants; dopaminergic antiparkinsonian agents (levodopa, dopamine agonists); ketamine and esketamine; classic psychedelics; antibacterial and antimycobacterial agents; TNF-α inhibitors; interferons; immune checkpoint inhibitors; montelukast; isotretinoin; varenicline; thyroid hormone; levetiracetam, baclofen, topiramate and zolpidem; herbal medicines
Concept block 3: design and data source	systematic review; meta-analysis; cohort; case–control; incidence; disproportionality; reporting odds ratio; pharmacovigilance; FAERS; VigiBase; EudraVigilance; JADER
Mechanistic sections	Imaging and microbiome terms combined with the outcome block for Sections 3.3.1–3.3.7

Screening	Title and abstract screening by a single reviewer (the author); full text examined when relevance or the outcome definition could not be determined from the abstract, including inspection of outcome definitions and results tables to establish whether mania or hypomania was a specified outcome
Inclusion criteria	(i) Manic or hypomanic states, or an outcome from which these could be separated, in association with exposure to, dose change in or withdrawal from a systemic therapeutic agent in humans, or with controlled experimental administration; (ii) neurobiology of one of the five proximal axes or of the network onto which they are proposed to converge, in humans or experimental models; (iii) nomenclature, measurement or pharmacovigilance methods
Exclusion criteria	Recreational substance use; focal neuromodulation (admitted only as corroborating evidence); mania secondary to medical illness, except where illness and treatment were difficult to separate
Evidence grading	Four design-specific grades (strong, moderate, indirect or single source, absent) applied separately within each study design (Section 2.1); cell-level sources in Supplementary Table S4; structured appraisal of studies informing strong or moderate grades in Supplementary Table S7
Limitations of the procedure	Not registered; no duplicate independent screening or extraction; no single risk-of-bias instrument; case-report literature not retrieved exhaustively; record counts at each screening stage not logged
