# Supplementary material for: From Pharmacological Trigger to Manic State: Convergent Neurobiological Mechanisms of Drug-Induced Mania
Source: Brain Sci. 2026 Oct 8;16(10):1081. doi: 10.3390/brainsci16101081 (PMC13702241; doi:10.3390/brainsci16101081)
Supplement: Supplementary file 1 [file brainsci-16-01081-s001.zip › Table S5.pdf]

Supplementary Material

From pharmacological trigger to manic state: convergent neurobiological mechanisms of drug-induced mania

Ji Hyun Park

College of Pharmacy, Duksung Women's University, Seoul, Republic of Korea

Table S5: Pooled and drug-level summaries of reported latency by mechanistic group and by mechanistic axis

The drug-level median is the median of per-drug medians among drugs contributing at least five drug–report pairs, so that each drug carries equal weight; intervals are percentile bootstrap intervals resampling drugs (5,000 replicates). Across the five principal plotting groups, drug-level medians did not differ significantly (Kruskal–Wallis test: all dated pairs, p = 0.16; complete dates, p = 0.10).

Dataset	Level	Group or axis	Drug–report pairs	Pooled median (IQR), days	Drugs with ≥5 pairs	Drug-level median (95% bootstrap interval), days
All dated pairs	Plotting group (Figure 5A)	Glutamatergic / rapid-acting	52	1 (0–23.8)	4	2 (0–3)
All dated pairs	Plotting group (Figure 5A)	Anti-infective / immune	314	4 (0–87)	22	10.5 (3–78)
All dated pairs	Plotting group (Figure 5A)	Glucocorticoid / endocrine	254	6 (0–29)	8	13.5 (1–28)
All dated pairs	Plotting group (Figure 5A)	Dopaminergic / stimulant	239	16 (0–181)	10	28 (2.5–60.5)
All dated pairs	Plotting group (Figure 5A)	Monoaminergic antidepressant	781	25 (0–211)	13	25 (13–31)
All dated pairs	Plotting group (Figure 5A)	Other CNS / metabolic	434	15.5 (0–119.8)	13	21 (17–42)
All dated pairs	Mechanistic axis	Dopamine agonists and levodopa	81	54 (0–476)	3	49 (26–233)
All dated pairs	Mechanistic axis	Psychostimulants	158	5 (0–74)	7	5 (0–60.5)
All dated pairs	Mechanistic axis	Monoaminergic antidepressants	781	25 (0–211)	13	25 (13–31)

All dated pairs	Mechanistic axis	Glutamatergic agents	52	1 (0–23.8)	4	2 (0–3)
All dated pairs	Mechanistic axis	Glucocorticoids	234	6 (0–23)	6	5.5 (0.5–18.8)
All dated pairs	Mechanistic axis	Testosterone and levothyroxine	20	30 (2.2–864)	2	125.8 (28–223.5)
All dated pairs	Mechanistic axis	Anti-infective agents	133	3 (1–6)	9	3 (1–6)
All dated pairs	Mechanistic axis	Immunomodulatory agents	181	20 (0–340)	13	69 (15–99)
All dated pairs	Mechanistic axis	Other CNS agents	411	14 (0–122.5)	11	21 (11–168)
All dated pairs	Mechanistic axis	Metabolic agents	23	29 (6.5–84.5)	2	24.5 (17–32)
Complete eight-digit dates	Plotting group (Figure 5A)	Glutamatergic / rapid-acting	46	2.5 (0–27.2)	4	2 (0–6.5)
Complete eight-digit dates	Plotting group (Figure 5A)	Anti-infective / immune	207	4 (1–40)	16	5.5 (2.8–27)
Complete eight-digit dates	Plotting group (Figure 5A)	Glucocorticoid / endocrine	193	6 (1–20)	6	13 (2.5–20.2)
Complete eight-digit dates	Plotting group (Figure 5A)	Dopaminergic / stimulant	147	16 (0–192)	8	15.8 (3–233)
Complete eight-digit dates	Plotting group (Figure 5A)	Monoaminergic antidepressant	470	20 (1–115)	13	20 (11–26)
Complete eight-digit dates	Plotting group (Figure 5A)	Other CNS / metabolic	230	11 (2–38.5)	13	11 (4–17)
Complete eight-digit dates	Mechanistic axis	Dopamine agonists and levodopa	40	329.5 (6.2–575)	2	340.5 (233–448)
Complete eight-digit dates	Mechanistic axis	Psychostimulants	107	8 (0–60.5)	6	4 (2.5–92.8)
Complete eight-digit dates	Mechanistic axis	Monoaminergic antidepressants	470	20 (1–115)	13	20 (11–26)
Complete eight-digit dates	Mechanistic axis	Glutamatergic agents	46	2.5 (0–27.2)	4	2 (0–6.5)
Complete eight-digit dates	Mechanistic axis	Glucocorticoids	183	6 (1–15.5)	5	8 (1–20)
Complete eight-digit dates	Mechanistic axis	Testosterone and levothyroxine	10	20.5 (0.8–92)	1	20.5
Complete eight-digit dates	Mechanistic axis	Anti-infective agents	112	3 (1–6)	8	3 (2–6)
Complete eight-digit dates	Mechanistic axis	Immunomodulatory agents	95	20 (1–158.5)	8	32.5 (3–86)
Complete eight-digit dates	Mechanistic axis	Other CNS agents	214	11 (2–34.5)	11	10 (2.5–17)
Complete eight-digit dates	Mechanistic axis	Metabolic agents	16	23.5 (7.8–81.8)	2	20.2 (17–23.5)

Table S5 (continued). Per-drug reported time to onset for drugs contributing at least five drug–report pairs (all dated pairs).

Plotting group	Mechanistic axis	Drug	Pairs	Median (IQR), days
Anti-infective / immune	Anti-infective agents	Metronidazole	11	0 (0–3)
Anti-infective / immune	Anti-infective agents	Clarithromycin	29	1 (0–1)
Anti-infective / immune	Anti-infective agents	Moxifloxacin	17	2 (1–13)
Anti-infective / immune	Anti-infective agents	Oseltamivir	6	2.5 (2–3.8)
Anti-infective / immune	Anti-infective agents	Levofloxacin	22	3 (1.5–3)
Anti-infective / immune	Anti-infective agents	Ciprofloxacin	19	4 (2–4)
Anti-infective / immune	Anti-infective agents	Azithromycin	5	6 (6–6)
Anti-infective / immune	Anti-infective agents	Hydroxychloroquine	6	6 (6–15.8)
Anti-infective / immune	Anti-infective agents	Isoniazid	5	87 (87–87)
Anti-infective / immune	Immunomodulatory agents	Ipilimumab	9	0 (0–86)
Anti-infective / immune	Immunomodulatory agents	Infliximab	6	1 (0–10.2)
Anti-infective / immune	Immunomodulatory agents	Montelukast	47	3 (0–431.5)
Anti-infective / immune	Immunomodulatory agents	Etanercept	6	15 (0–76.5)
Anti-infective / immune	Immunomodulatory agents	Interferon alfa	6	17.5 (9.5–20.2)
Anti-infective / immune	Immunomodulatory agents	Nivolumab	12	32 (0–101.5)
Anti-infective / immune	Immunomodulatory agents	Adalimumab	30	69 (1–752.5)
Anti-infective / immune	Immunomodulatory agents	Dupilumab	7	92 (30.5–209.5)
Anti-infective / immune	Immunomodulatory agents	Isotretinoin	7	92 (9.5–129)
Anti-infective / immune	Immunomodulatory agents	Fingolimod	13	99 (48–356)
Anti-infective / immune	Immunomodulatory agents	Interferon beta	7	104 (38–1516)
Anti-infective / immune	Immunomodulatory agents	Dimethyl fumarate	8	236.5 (9.5–629.5)
Anti-infective / immune	Immunomodulatory agents	Natalizumab	9	1480 (0–1836)
Dopaminergic / stimulant	Dopamine agonists and levodopa	Levodopa	45	26 (0–544)
Dopaminergic / stimulant	Dopamine agonists and levodopa	Pramipexole	11	49 (7–69.5)
Dopaminergic / stimulant	Dopamine agonists and levodopa	Ropinirole	9	233 (54–438)

Dopaminergic / stimulant	Psychostimulants	Amphetamine	19	0 (0–46)
Dopaminergic / stimulant	Psychostimulants	Methylphenidate	56	0 (0–31.2)
Dopaminergic / stimulant	Psychostimulants	Solriamfetol	5	3 (0–8)
Dopaminergic / stimulant	Psychostimulants	Atomoxetine	20	5 (0–32)
Dopaminergic / stimulant	Psychostimulants	Viloxazine	13	30 (17–45)
Dopaminergic / stimulant	Psychostimulants	Lisdexamfetamine	30	60.5 (0–192)
Dopaminergic / stimulant	Psychostimulants	Phentermine	8	65 (0–90.2)
Glucocorticoid / endocrine	Glucocorticoids	Hydrocortisone	7	0 (0–121.5)
Glucocorticoid / endocrine	Glucocorticoids	Methylprednisolone	39	1 (0–11)
Glucocorticoid / endocrine	Glucocorticoids	Prednisolone	75	2 (0–6.5)
Glucocorticoid / endocrine	Glucocorticoids	Prednisone	61	9 (2–28)
Glucocorticoid / endocrine	Glucocorticoids	Triamcinolone	6	18 (5.2–41.2)
Glucocorticoid / endocrine	Glucocorticoids	Dexamethasone	42	19.5 (3.8–59)
Glucocorticoid / endocrine	Testosterone and levothyroxine	Levothyroxine	8	28 (2.2–43.8)
Glucocorticoid / endocrine	Testosterone and levothyroxine	Testosterone	12	223.5 (9–864)
Glutamatergic / rapid-acting	Glutamatergic agents	Zuranolone	7	0 (0–3.5)
Glutamatergic / rapid-acting	Glutamatergic agents	Ketamine	9	1 (0–64)
Glutamatergic / rapid-acting	Glutamatergic agents	Dextromethorphan	9	3 (0–9)
Glutamatergic / rapid-acting	Glutamatergic agents	Esketamine	25	3 (0–135)
Monoaminergic antidepressant	Monoaminergic antidepressants	Sertraline	140	4 (0–115)
Monoaminergic antidepressant	Monoaminergic antidepressants	Bupropion	42	5 (0–35)
Monoaminergic antidepressant	Monoaminergic antidepressants	Mirtazapine	43	12 (1–38)
Monoaminergic antidepressant	Monoaminergic antidepressants	Desvenlafaxine	18	13 (0.2–49)
Monoaminergic antidepressant	Monoaminergic antidepressants	Amitriptyline	10	17.5 (0–1009.5)
Monoaminergic antidepressant	Monoaminergic antidepressants	Citalopram	36	24 (4–211)
Monoaminergic antidepressant	Monoaminergic antidepressants	Fluoxetine	49	25 (0–87)

Monoaminergic antidepressant	Monoaminergic antidepressants	Vortioxetine	50	26.5 (2–45.2)
Monoaminergic antidepressant	Monoaminergic antidepressants	Duloxetine	119	30 (1–179)
Monoaminergic antidepressant	Monoaminergic antidepressants	Venlafaxine	102	31 (0–681)
Monoaminergic antidepressant	Monoaminergic antidepressants	Trazodone	12	38 (0.8–154.5)
Monoaminergic antidepressant	Monoaminergic antidepressants	Escitalopram	73	41 (0–1096)
Monoaminergic antidepressant	Monoaminergic antidepressants	Paroxetine	75	606 (26–1249)
Other CNS / metabolic	Metabolic agents	Semaglutide	9	17 (1–36)
Other CNS / metabolic	Metabolic agents	Tirzepatide	12	32 (15.8–81.8)
Other CNS / metabolic	Other CNS agents	Tetrabenazine	36	6.5 (0–26)
Other CNS / metabolic	Other CNS agents	Lamotrigine	79	7 (0–53.5)
Other CNS / metabolic	Other CNS agents	Topiramate	49	11 (9–29)
Other CNS / metabolic	Other CNS agents	Pregabalin	31	17 (0–95.5)
Other CNS / metabolic	Other CNS agents	Zolpidem	27	19 (0–176)
Other CNS / metabolic	Other CNS agents	Levetiracetam	36	21 (0–39)
Other CNS / metabolic	Other CNS agents	Varenicline	19	31 (20–50.5)
Other CNS / metabolic	Other CNS agents	Sodium oxybate	57	42 (0–365)
Other CNS / metabolic	Other CNS agents	Tramadol	27	168 (2–2922)
Other CNS / metabolic	Other CNS agents	Gabapentin	35	183 (1.5–445.5)
Other CNS / metabolic	Other CNS agents	Baclofen	15	184 (1.5–308)
