# Supplementary material for: From Pharmacological Trigger to Manic State: Convergent Neurobiological Mechanisms of Drug-Induced Mania
Source: Brain Sci. 2026 Oct 8;16(10):1081. doi: 10.3390/brainsci16101081 (PMC13702241; doi:10.3390/brainsci16101081)
Supplement: Supplementary file 1 [file brainsci-16-01081-s001.zip › Table S4.pdf]

Supplementary Material

From pharmacological trigger to manic state: convergent neurobiological mechanisms of drug-induced mania

Ji Hyun Park

College of Pharmacy, Duksung Women’s University, Seoul, Republic of Korea

Table S4: Study-design-specific evidence grades underlying Figure 3, with the sources for each cell

Reference numbers refer to the reference list of the main text. Cells changed to “absent” in this revision are listed explicitly; other cells not listed are also graded “absent” (no evidence of that design identified in the searches). “Null” marks cells in which the specified-outcome evidence did not show an excess of mania.

Drug class	Study design	Grade	Basis	References
Corticosteroids	Randomized or experimental	Indirect	Meta-analysis pooling studies of mixed design on psychiatric symptoms; randomized component not separable for mania	[70]
Corticosteroids	Cohort, case–control or self-controlled	Strong	Population cohort with same-disease unexposed comparators (mania HR 4.35) and meta-analysis of 73 studies with manic symptoms as an outcome	[9,70]
Corticosteroids	Systematic review of case material	Indirect	Systematic review of case-based literature	[2]
Corticosteroids	Case reports or case series	Indirect	Case series	[54,71]
Anabolic-androgenic steroids	Randomized or experimental	Strong	Four controlled administration studies with manic or hypomanic symptoms rated; findings inconsistent; replacement-dose trial without mania signal	[56,59–62]

Anabolic-androgenic steroids	Cohort, case–control or self-controlled	Moderate	Single controlled comparison of steroid users and nonusers with manic syndromes ascertained by structured interview	[63]
Anabolic-androgenic steroids	Systematic review of case material	Indirect	PRISMA systematic review of 37 studies of psychopathology in steroid-using athletes, including eight case reports; hypomania described with short-term use, but mania not analyzed separately	[65]
Anabolic-androgenic steroids	Case reports or case series	Indirect	Uncontrolled interview series of 41 users	[64]
Dopaminergic antiparkinsonian agents	Randomized or experimental	Moderate	Single experimental levodopa challenge with hypomania as the outcome	[66]
Dopaminergic antiparkinsonian agents	Cohort, case–control or self-controlled	Indirect	Large cross-sectional study quantifying impulse-control disorders rather than mania	[5]
Dopaminergic antiparkinsonian agents	Systematic review of case material	Indirect	Systematic review of published dopamine dysregulation syndrome cases	[113]
Dopaminergic antiparkinsonian agents	Case reports or case series	Indirect	Cases pooled in the systematic review	[113]
Psychostimulants	Randomized or experimental	Strong (null)	Pooled analysis of five randomized studies with manic symptoms as an outcome; no effect	[4]
Psychostimulants	Cohort, case–control or self-controlled	Strong	Within-individual register study and replications with mania as the outcome	[10,72,73]
Psychostimulants	Systematic review of case material	Absent (changed in revision)	No source of this design identified in the searches	–
Psychostimulants	Case reports or case series	Indirect	Case report of mania after methylphenidate initiation in an adult with ADHD, with actigraphic sleep reduction preceding the episode	[133]
Antidepressants	Randomized or experimental	Strong (null)	Network meta-analysis of 13 randomized trials in bipolar depression; no agent above placebo	[3]
Antidepressants	Cohort, case–control or self-controlled	Strong	Target trial emulation (null) and systematic review of 32 naturalistic studies (17.3–48.8%)	[67,68]
Antidepressants	Systematic review of case	Absent (changed in	No source of this design identified in the searches (the review previously coded here concerns discontinuation states and is	–

	material	revision)	retained under Withdrawal states)	
Antidepressants	Case reports or case series	Indirect	Case report of mania after sertraline initiation in a patient without personal or family history of bipolar disorder; no recurrence over two years	[69]
Ketamine / esketamine	Randomized or experimental	Indirect	Switch events pooled across eight studies, captured as adverse events rather than by systematic assessment	[6]
Ketamine / esketamine	Cohort, case–control or self-controlled	Moderate	Single propensity-matched cohort with manic switch as the outcome; one uncontrolled service cohort	[77,78]
Ketamine / esketamine	Systematic review of case material	Absent (changed in revision)	No source of this design identified in the searches	–
Ketamine / esketamine	Case reports or case series	Indirect	Case report of switch to mania during subanesthetic ketamine for unipolar treatment-resistant depression	[121]
Classic psychedelics	Randomized or experimental	Moderate	Supervised trials pooled in a meta-analysis; mania recorded but not the trials' specified outcome	[19]
Classic psychedelics	Cohort, case–control or self-controlled	Moderate	Naturalistic samples of individuals with bipolar disorder pooled in the same meta-analysis	[19]
Classic psychedelics	Systematic review of case material	Indirect	Systematic review of case reports: 17 affective episodes, including mania, in individuals without previous major psychiatric disorder	[82]
Classic psychedelics	Case reports or case series	Indirect	Case report of mania after psilocybin ingestion in a woman without a prior bipolar diagnosis	[83]
Antibiotics and antimycobacterials	Cohort, case–control or self-controlled	Indirect	Adolescent doxycycline cohort (later manic or bipolar diagnosis, judged non-causal); case–control study unable to separate antibiotic from infection	[79,155]
Antibiotics and antimycobacterials	Systematic review of case material	Indirect	Systematic reviews and spontaneous-report reviews of case material	[7,17,154]
Antibiotics and antimycobacterials	Case reports or case series	Indirect	Case reports	[158,178]
TNF-α inhibitors	Cohort, case–control or self-controlled	Indirect	Single cohort retained within a systematic review, reporting elated mood (40 of 7,600 patients) rather than a defined manic or hypomanic outcome	[8]
TNF-α inhibitors	Systematic review of case	Indirect	Systematic review	[8]

	material			
TNF-α inhibitors	Case reports or case series	Indirect	Case reports pooled in the systematic review	[8]
Interferons	Cohort, case–control or self-controlled	Moderate	Single prospective cohort with systematic assessment of manic and hypomanic states	[38]
Interferons	Systematic review of case material	Absent (changed in revision)	No source of this design identified in the searches	–
Interferons	Case reports or case series	Indirect	Case series	[81]
Immune checkpoint inhibitors	Case reports or case series	Absent (changed in revision)	No source of this design identified in the searches; the only quantitative resource is a disproportionality analysis that does not isolate mania	[104]
Montelukast	Cohort, case–control or self-controlled	Moderate (null)	Single propensity-matched cohort with a prespecified manic outcome (no excess); national cohort with a composite excluding mania	[87,88]
Montelukast	Systematic review of case material	Absent (changed in revision)	No source of this design identified in the searches; a systematic review of neuropsychiatric events with montelukast contained no manic or hypomanic case	–
Montelukast	Case reports or case series	Indirect	Case report of hypomania one week after montelukast in a woman with long-remitted bipolar disorder; resolution after discontinuation	[89]
Isotretinoin	Cohort, case–control or self-controlled	Indirect	Cohorts addressing psychiatric composites without a mania-specific estimate	[90,93]
Isotretinoin	Systematic review of case material	Indirect	Structured literature review of case reports, case series and registries concluding that many states recorded as depressive had manic or mixed features	[92]
Isotretinoin	Case reports or case series	Indirect	Case report of psychotic mania within the first week of treatment in a young man without personal or family psychiatric history	[91]
Thyroid hormone	Case reports or case series	Indirect	Case report	[103]
Other CNS agents	Cohort, case–control or self-controlled	Absent (changed in revision)	No source of this design identified in the searches	–
Other CNS agents	Systematic review of case material	Indirect	Systematic review of published levetiracetam cases with formal causality appraisal	[85]

Other CNS agents	Case reports or case series	Indirect	Cases pooled in the systematic review	[85]
Withdrawal states	Cohort, case–control or self-controlled	Moderate (null)	Single self-controlled case series of glucocorticoid discontinuation with mania prespecified; no excess	[106]
Withdrawal states	Systematic review of case material	Indirect	Review of antidepressant discontinuation manic states	[105]
Withdrawal states	Case reports or case series	Indirect	Cases pooled in the review	[105]

Case-level evidence (case reports, case series and systematic reviews of case material) lacks an exposure denominator and is therefore not graded above "indirect or single source" (Section 2.1).
