# Supplementary material for: From Pharmacological Trigger to Manic State: Convergent Neurobiological Mechanisms of Drug-Induced Mania
Source: Brain Sci. 2026 Oct 8;16(10):1081. doi: 10.3390/brainsci16101081 (PMC13702241; doi:10.3390/brainsci16101081)
Supplement: Supplementary file 1 [file brainsci-16-01081-s001.zip › Table S3.pdf]

Supplementary Material

From pharmacological trigger to manic state: convergent neurobiological mechanisms of drug-induced mania

Ji Hyun Park

College of Pharmacy, Duksung Women's University, Seoul, Republic of Korea

Table S3: Definition and computation of the temporal-concentration flag

Definition. For each agent, the share of its manic or hypomanic reports falling in its peak calendar year was divided by the share of all reports for that agent falling in the same year. A ratio near 1 indicates that manic reports follow the agent's own reporting history; values well above 1 indicate disproportionate clustering of manic reports in a single year. The ratio is a descriptive flag, not a validated diagnostic: it cannot distinguish litigation, batch submission, publicity, product launch or changes in market share, and it is unstable below about ten manic reports (Supplementary Methods S1, Section S1.9).

For every agent with at least three manic reports: the share of manic reports in the peak calendar year, the share of all reports for that agent in the same year, and their ratio. Also provided as temporal_concentration.csv in Code S1.

Agent	Axis	Manic reports	All reports	Peak year	Manic-report share in peak year	All-report share in same year	Ratio
Losartan	CTRL	3	15,283	2017	1.000	0.063	15.84
Ropinirole	DA	18	2,290	2023	0.222	0.027	8.21
Isoniazid	ABX	7	2,719	2022	0.429	0.056	7.72
Oseltamivir	ABX	19	9,368	2021	0.316	0.043	7.30
Amlodipine	CTRL	11	46,920	2024	0.455	0.068	6.65
Certolizumab	IMM	6	78,465	2022	0.333	0.051	6.58

Fluvoxamine	AD	4	946	2018	0.500	0.086	5.84
Testosterone	HORM	23	31,379	2024	0.217	0.039	5.55
Levocetirizine	CTRL	22	39,960	2022	0.409	0.074	5.55
Metronidazole	ABX	37	14,618	2023	0.351	0.066	5.36
Nortriptyline	AD	6	1,175	2021	0.500	0.095	5.25
Acyclovir	ABX	4	15,281	2024	0.500	0.097	5.15
Duloxetine	AD	802	35,007	2017	0.623	0.124	5.02
Chloroquine	ABX	9	325	2017	0.778	0.157	4.96
Pembrolizumab	IMM	3	59,688	2018	0.333	0.071	4.72
Tocilizumab	IMM	6	54,700	2025	0.333	0.073	4.55
Rotigotine	DA	4	7,348	2016	0.250	0.057	4.37
Phenelzine	AD	6	457	2023	0.333	0.077	4.35
Apomorphine	DA	6	7,819	2018	0.500	0.117	4.28
Azithromycin	ABX	22	19,295	2025	0.364	0.085	4.27
Levofloxacin	ABX	37	25,694	2025	0.216	0.051	4.25
Amitriptyline	AD	13	4,018	2021	0.385	0.093	4.14
Hydroxychloroquine	ABX	5	15,937	2020	0.800	0.203	3.95
Memantine	GLU	27	4,273	2024	0.259	0.066	3.93
Clomipramine	AD	12	813	2017	0.250	0.065	3.83
Baclofen	CNS	79	16,830	2015	0.443	0.118	3.74
Selegiline	DA	3	560	2015	0.667	0.179	3.73
Imipramine	AD	3	420	2018	0.667	0.179	3.73
Efavirenz	ABX	7	12,119	2016	0.286	0.078	3.64
Desvenlafaxine	AD	41	6,825	2019	0.341	0.098	3.48
Phentermine	STIM	8	6,443	2015	0.250	0.072	3.48
Teriflunomide	IMM	6	36,330	2020	0.333	0.103	3.24
Natalizumab	IMM	17	58,405	2015	0.412	0.127	3.24
Trazodone	AD	18	4,405	2017	0.222	0.071	3.14
Risperidone	PSY	171	61,153	2025	0.152	0.050	3.05
Cabergoline	DA	19	1,502	2016	0.263	0.087	3.04
Ketamine	GLU	32	1,621	2021	0.312	0.109	2.86
Metformin	CTRL	5	73,310	2017	0.200	0.073	2.72
Hydrocortisone	GC	21	9,249	2023	0.333	0.125	2.67
Moxifloxacin	ABX	22	6,550	2022	0.182	0.070	2.59
Montelukast	IMM	64	14,523	2024	0.344	0.133	2.59
Paroxetine	AD	116	12,148	2016	0.336	0.132	2.55
Etanercept	IMM	10	283,328	2016	0.500	0.198	2.53
Armodafinil	STIM	8	1,479	2015	0.500	0.201	2.49

Infliximab	IMM	17	124,275	2025	0.235	0.095	2.49
Ustekinumab	IMM	8	73,490	2023	0.250	0.104	2.41
Sodium oxybate	CNS	142	66,044	2024	0.225	0.094	2.40
Tramadol	CNS	86	30,960	2015	0.174	0.073	2.39
Pseudoephedrine	STIM	11	14,012	2017	0.364	0.156	2.33
Pramipexole	DA	48	3,923	2015	0.229	0.102	2.24
Interferon beta	IMM	12	79,340	2018	0.250	0.118	2.12
Zolpidem	CNS	52	14,933	2017	0.173	0.084	2.07
Topiramate	CNS	79	20,443	2015	0.241	0.117	2.06
Isotretinoin	IMM	22	34,801	2021	0.182	0.090	2.03
Liraglutide	MET	3	21,015	2015	0.333	0.169	1.97
Dextromethorphan	GLU	47	22,692	2016	0.170	0.087	1.97
Varenicline	CNS	53	17,542	2019	0.264	0.139	1.90
Fingolimod	IMM	16	67,647	2016	0.250	0.133	1.88
Clarithromycin	ABX	79	11,063	2020	0.190	0.101	1.88
Budesonide	GC	22	50,071	2023	0.227	0.121	1.88
Istradefylline	DA	10	5,047	2020	0.500	0.269	1.86
Amphetamine	STIM	49	20,027	2021	0.163	0.089	1.84
Adalimumab	IMM	54	451,372	2023	0.167	0.091	1.83
Dimethyl fumarate	IMM	14	78,217	2015	0.286	0.157	1.82
Modafinil	STIM	14	1,800	2016	0.214	0.118	1.81
Levetiracetam	CNS	155	47,576	2019	0.239	0.134	1.78
Mirtazapine	AD	103	13,505	2018	0.184	0.105	1.75
Escitalopram	AD	194	16,282	2022	0.144	0.084	1.72
Dexamethasone	GC	94	43,442	2024	0.191	0.111	1.72
Levodopa	DA	98	69,663	2019	0.184	0.108	1.71
Dupilumab	IMM	21	430,295	2023	0.238	0.141	1.69
Nivolumab	IMM	9	68,212	2019	0.222	0.133	1.67
Solriamfetol	STIM	7	2,060	2022	0.429	0.266	1.61
Triamcinolone	GC	10	15,408	2015	0.200	0.126	1.59
Methylphenidate	STIM	124	28,449	2023	0.137	0.088	1.55
Omeprazole	CTRL	20	30,751	2020	0.200	0.130	1.54
Vortioxetine	AD	149	13,147	2019	0.174	0.113	1.54
Semaglutide	MET	22	60,839	2025	0.545	0.355	1.54
Bupropion	AD	158	32,363	2024	0.209	0.137	1.53
Methylprednisolone	GC	78	21,037	2020	0.192	0.128	1.50
Levothyroxine	HORM	22	44,046	2017	0.273	0.185	1.47
Citalopram	AD	117	16,312	2018	0.162	0.111	1.47

Prednisone	GC	90	37,734	2023	0.144	0.100	1.45
Aripiprazole	PSY	882	51,050	2017	0.274	0.190	1.44
Venlafaxine	AD	258	27,285	2019	0.186	0.134	1.39
Lamotrigine	CNS	226	28,885	2015	0.159	0.115	1.39
Sertraline	AD	387	38,445	2018	0.129	0.094	1.38
Tirzepatide	MET	31	133,100	2025	0.581	0.439	1.32
Quetiapine	PSY	363	39,209	2023	0.118	0.093	1.28
Ciprofloxacin	ABX	48	29,334	2019	0.188	0.147	1.27
Lithium	PSY	78	4,110	2018	0.154	0.122	1.26
Olanzapine	PSY	355	35,036	2015	0.225	0.189	1.19
Gabapentin	CNS	72	50,222	2019	0.208	0.177	1.18
Pregabalin	CNS	64	70,176	2019	0.156	0.140	1.12
Lisdexamfetamine	STIM	90	15,220	2023	0.133	0.120	1.11
Prednisolone	GC	146	32,461	2018	0.158	0.143	1.10
Tetrabenazine	CNS	94	34,095	2018	0.149	0.136	1.10
Valproate	PSY	147	19,191	2021	0.156	0.149	1.05
Atomoxetine	STIM	71	6,714	2015	0.634	0.620	1.02
Viloxazine	STIM	22	857	2025	0.273	0.292	0.93
Zuranolone	GLU	14	1,315	2025	0.500	0.567	0.88
Esketamine	GLU	74	16,906	2025	0.311	0.360	0.86
Fluoxetine	AD	168	18,795	2015	0.155	0.183	0.85
Interferon alfa	IMM	23	24,080	2015	0.522	0.747	0.70

Agents are sorted by ratio. Axis codes: ABX, anti-infective; IMM, immunomodulatory; STIM, psychostimulant; DA, dopamine agonist or levodopa; GC, glucocorticoid; HORM, androgen or thyroid hormone; GLU, glutamatergic; AD, monoaminergic antidepressant; CNS, other central nervous system agent; MET, metabolic agent; PSY, psychotropic indication comparator; CTRL, negative control.
