# Supplementary material for: From Pharmacological Trigger to Manic State: Convergent Neurobiological Mechanisms of Drug-Induced Mania
Source: Brain Sci. 2026 Oct 8;16(10):1081. doi: 10.3390/brainsci16101081 (PMC13702241; doi:10.3390/brainsci16101081)
Supplement: Supplementary file 1 [file brainsci-16-01081-s001.zip › Table S2.pdf]

Supplementary Material

From pharmacological trigger to manic state: convergent neurobiological mechanisms of drug-induced mania

Ji Hyun Park

College of Pharmacy, Duksung Women's University, Seoul, Republic of Korea

Table S2: Time-to-onset analysis restricted to reports carrying complete eight-digit start and event dates

Median interval from recorded therapy start to event, by mechanistic group, comparing all dated pairs (partial dates completed to the first day of the stated month or year) with an analysis restricted to records carrying complete eight-digit dates for both fields.

Mechanistic group	All dated pairs, n	All dated pairs, median (days)	Complete dates, n	Complete-date median (days)
Glutamatergic / rapid-acting	52	1	46	2.5
Anti-infective / immune	314	4	207	4
Glucocorticoid / endocrine	254	6	193	6
Other CNS / metabolic	434	15.5	230	11
Dopaminergic /	239	16	147	16

Mechanistic group	All dated pairs, n	All dated pairs, median (days)	Complete dates, n	Complete-date median (days)
stimulant				
Monoaminergic antidepressant	781	25	470	20
Indication comparator	667	7	304	7
Negative control	47	5	32	4

The ordering across mechanistic groups is preserved. The underlying drug–interval pairs are provided as time_to_onset_completedates.csv in Code S1.
