# Supplementary material for: From Pharmacological Trigger to Manic State: Convergent Neurobiological Mechanisms of Drug-Induced Mania
Source: Brain Sci. 2026 Oct 8;16(10):1081. doi: 10.3390/brainsci16101081 (PMC13702241; doi:10.3390/brainsci16101081)
Supplement: Supplementary file 1 [file brainsci-16-01081-s001.zip › Supplementary Methods S1.pdf]

Supplementary Material

From pharmacological trigger to manic state: convergent neurobiological mechanisms of drug-induced mania

Ji Hyun Park

College of Pharmacy, Duksung Women's University, Seoul, Republic of Korea

Supplementary Methods S1: Full description of the data source, ingredient mapping dictionary, deduplication procedure, disproportionality computation, and all sensitivity analyses

S1.1 Data source and period

All quarterly ASCII extracts of the United States Food and Drug Administration Adverse Event Reporting System (FAERS) from 2015Q1 through 2026Q1 (45 quarters) were downloaded from the agency's public distribution site. The FDA renamed the public system the Adverse Event Monitoring System (AEMS) on 11 March 2026; the 2026Q1 extract used here was posted on 28 April 2026 and retains the legacy `faers_ascii_` naming and the seven-table structure (DEMO, DRUG, REAC, INDI, THER, OUTC, RPSR), so the data as analyzed are unchanged. The DEMO, DRUG, REAC, INDI and THER files were used. The extracts contained 18,159,607 report records before deduplication.

S1.2 Case handling and deduplication

Case identifiers appearing in the quarterly deletion files (225,016 unique case identifiers) were removed. For each remaining case identifier, the record with the highest case version was retained, ties being broken by the FDA receipt date and then by the primary identifier,

leaving **15,419,124 unique cases**. Where the same primary identifier appeared unchanged in more than one quarterly file, only its first occurrence was retained.

S1.3 Outcome definition

The primary outcome was a report carrying the MedDRA preferred term *Mania* or *Hypomania* (11,698 cases). A broader definition additionally including *Bipolar disorder*, *Bipolar I disorder*, *Bipolar II disorder*, *Bipolar mania* and *Manic thoughts* (18,503 cases) was analyzed as a sensitivity analysis. No verbatim-term recoding was performed.

S1.4 Exposure definition

One hundred and seventeen active ingredients were specified in advance to populate the mechanistic axes of Section 3.3 of the main text, together with six pharmacologically unrelated agents (atorvastatin, amlodipine, omeprazole, metformin, losartan, levocetirizine (dictionary entry also matching cetirizine)) included as negative controls. Each drug record was matched to the ingredient dictionary by regular expression against the concatenated prod_ai (active ingredient) and drugname fields, upper-cased, with negative lookbehind assertions to prevent substring collisions (for example, AMPHETAMINE excluding LISDEXAMFETAMINE and METHAMPHETAMINE; CITALOPRAM excluding ESCITALOPRAM; KETAMINE excluding ESKETAMINE). The primary analysis counted a case as exposed when a matched ingredient was recorded with role code PS (primary suspect); a secondary analysis used PS or SS (secondary suspect). Matches were accumulated into a per-report set, so that each case contributed at most once to a given active ingredient regardless of how many drug records named it. The dictionary therefore contains 123 ingredients in total: 117 target agents and six negative controls. The resulting dictionary, including every regular expression, is listed below (the same dictionary is provided as drugs.py in Code S1).

Ingredient mapping dictionary

The 123 ingredients, their mechanistic axis and the regular expression used to match each ingredient against the upper-cased prod_ai and drugname fields are listed below in the order of the analysis code.

No.	Ingredient	Mechanistic axis	Regular expression
1	Levodopa	DA (Dopaminergic gain)	LEVODOPA
2	Pramipexole	DA (Dopaminergic gain)	PRAMIPEXOLE
3	Ropinirole	DA (Dopaminergic gain)	ROPINIROLE
4	Rotigotine	DA (Dopaminergic gain)	ROTIGOTINE
5	Apomorphine	DA (Dopaminergic gain)	APOMORPHINE
6	Amantadine	DA (Dopaminergic gain)	AMANTADINE
7	Selegiline	DA (Dopaminergic gain)	SELEGILINE
8	Rasagiline	DA (Dopaminergic gain)	RASAGILINE
9	Safinamide	DA (Dopaminergic gain)	SAFINAMIDE
10	Bromocriptine	DA (Dopaminergic gain)	BROMOCRIPTINE
11	Cabergoline	DA (Dopaminergic gain)	CABERGOLINE
12	Istradefylline	DA (Dopaminergic gain)	ISTRADEFYLLINE
13	Methylphenidate	STIM (Monoaminergic/stimulant)	METHYLPHENIDATE
14	Amphetamine	STIM (Monoaminergic/stimulant)	(?<!LISDEX)(?<!METH)AMPHETAMINE
15	Lisdexamfetamine	STIM (Monoaminergic/stimulant)	LISDEXAMFETAMINE|LISDEXAMPHETAMINE
16	Modafinil	STIM (Monoaminergic/stimulant)	\bMODAFINIL
17	Armodafinil	STIM (Monoaminergic/stimulant)	ARMODAFINIL
18	Atomoxetine	STIM (Monoaminergic/stimulant)	ATOMOXETINE
19	Viloxazine	STIM (Monoaminergic/stimulant)	VILOXAZINE
20	Solriamfetol	STIM (Monoaminergic/stimulant)	SOLRIAMFETOL
21	Phentermine	STIM (Monoaminergic/stimulant)	PHENTERMINE
22	Pseudoephedrine	STIM (Monoaminergic/stimulant)	PSEUDOEPHEDRINE
23	Fluoxetine	AD (Antidepressant (monoaminergic))	FLUOXETINE
24	Sertraline	AD (Antidepressant (monoaminergic))	SERTRALINE
25	Paroxetine	AD (Antidepressant (monoaminergic))	PAROXETINE
26	Citalopram	AD (Antidepressant (monoaminergic))	(?<!ES)CITALOPRAM
27	Escitalopram	AD (Antidepressant (monoaminergic))	ESCITALOPRAM
28	Fluvoxamine	AD (Antidepressant (monoaminergic))	FLUVOXAMINE
29	Venlafaxine	AD (Antidepressant (monoaminergic))	(?<!DES)VENLAFAXINE
30	Desvenlafaxine	AD (Antidepressant (monoaminergic))	DESVENLAFAXINE
31	Duloxetine	AD (Antidepressant (monoaminergic))	DULOXETINE
32	Bupropion	AD (Antidepressant (monoaminergic))	BUPROPION
33	Mirtazapine	AD (Antidepressant (monoaminergic))	MIRTAZAPINE
34	Vortioxetine	AD (Antidepressant (monoaminergic))	VORTIOXETINE
35	Amitriptyline	AD (Antidepressant (monoaminergic))	AMITRIPTYLINE
36	Nortriptyline	AD (Antidepressant (monoaminergic))	NORTRIPTYLINE
37	Imipramine	AD (Antidepressant (monoaminergic))	(?<!DES)IMIPRAMINE
38	Clomipramine	AD (Antidepressant (monoaminergic))	CLOMIPRAMINE
39	Tranylcypromine	AD (Antidepressant (monoaminergic))	TRANYLCYPROMINE
40	Phenelzine	AD (Antidepressant (monoaminergic))	PHENELZINE
41	Trazodone	AD (Antidepressant (monoaminergic))	TRAZODONE
42	Ketamine	GLU (Glutamatergic / rapid-acting)	(?<!ES)KETAMINE
43	Esketamine	GLU (Glutamatergic / rapid-acting)	ESKETAMINE
44	Dextromethorphan	GLU (Glutamatergic / rapid-acting)	DEXTROMETHORPHAN
45	Memantine	GLU (Glutamatergic / rapid-acting)	MEMANTINE
46	Zuranolone	GLU (Glutamatergic / rapid-acting)	ZURANOLONE
47	Brexanolone	GLU (Glutamatergic / rapid-acting)	BREXANOLONE
48	Psilocybin	SER (Serotonergic psychedelic)	PSILOCYBIN
49	Lysergide	SER (Serotonergic psychedelic)	LYSERGIDE|LYSERGIC ACID
50	Prednisone	GC (Glucocorticoid / HPA)	\bPREDNISONE
51	Prednisolone	GC (Glucocorticoid / HPA)	(?<!METHYL)PREDNISOLONE
52	Methylprednisolone	GC (Glucocorticoid / HPA)	METHYLPREDNISOLONE
53	Dexamethasone	GC (Glucocorticoid / HPA)	DEXAMETHASONE
54	Hydrocortisone	GC (Glucocorticoid / HPA)	HYDROCORTISONE
55	Budesonide	GC (Glucocorticoid / HPA)	BUDESONIDE
56	Triamcinolone	GC (Glucocorticoid / HPA)	TRIAMCINOLONE
57	Levothyroxine	HORM (Other endocrine)	LEVOTHYROXINE|THYROXINE
58	Liothyronine	HORM (Other endocrine)	LIOTHYRONINE
59	Testosterone	HORM (Other endocrine)	TESTOSTERONE
60	Oxandrolone	HORM (Other endocrine)	OXANDROLONE

61	Clarithromycin	ABX (Anti-infective)	CLARITHROMYCIN
62	Azithromycin	ABX (Anti-infective)	AZITHROMYCIN
63	Erythromycin	ABX (Anti-infective)	(?<!CLARITH)(?<!AZITH)ERYTHROMYCIN
64	Levofloxacin	ABX (Anti-infective)	LEVOFLOXACIN
65	Ciprofloxacin	ABX (Anti-infective)	CIPROFLOXACIN
66	Moxifloxacin	ABX (Anti-infective)	MOXIFLOXACIN
67	Isoniazid	ABX (Anti-infective)	ISONIAZID
68	Cycloserine	ABX (Anti-infective)	CYCLOSERINE
69	Mefloquine	ABX (Anti-infective)	MEFLOQUINE
70	Hydroxychloroquine	ABX (Anti-infective)	HYDROXYCHLOROQUINE
71	Chloroquine	ABX (Anti-infective)	(?<!HYDROXY)CHLOROQUINE
72	Efavirenz	ABX (Anti-infective)	EFAVIRENZ
73	Oseltamivir	ABX (Anti-infective)	OSELTAMIVIR
74	Metronidazole	ABX (Anti-infective)	METRONIDAZOLE
75	Linezolid	ABX (Anti-infective)	LINEZOLID
76	Cefepime	ABX (Anti-infective)	CEFEPIME
77	Acyclovir	ABX (Anti-infective)	ACICLOVIR|ACYCLOVIR
78	Adalimumab	IMM (Immune / cytokine)	ADALIMUMAB
79	Etanercept	IMM (Immune / cytokine)	ETANERCEPT
80	Infliximab	IMM (Immune / cytokine)	INFLIXIMAB
81	Golimumab	IMM (Immune / cytokine)	GOLIMUMAB
82	Certolizumab	IMM (Immune / cytokine)	CERTOLIZUMAB
83	Interferon beta	IMM (Immune / cytokine)	INTERFERON BETA
84	Interferon alfa	IMM (Immune / cytokine)	INTERFERON ALFA|PEGINTERFERON ALFA
85	Tocilizumab	IMM (Immune / cytokine)	TOCILIZUMAB
86	Ustekinumab	IMM (Immune / cytokine)	USTEKINUMAB
87	Dupilumab	IMM (Immune / cytokine)	DUPILUMAB
88	Montelukast	IMM (Immune / cytokine)	MONTELUKAST
89	Isotretinoin	IMM (Immune / cytokine)	ISOTRETINOIN
90	Natalizumab	IMM (Immune / cytokine)	NATALIZUMAB
91	Fingolimod	IMM (Immune / cytokine)	FINGOLIMOD
92	Dimethyl fumarate	IMM (Immune / cytokine)	DIMETHYL FUMARATE
93	Teriflunomide	IMM (Immune / cytokine)	TERIFLUNOMIDE
94	Pembrolizumab	IMM (Immune / cytokine)	PEMBROLIZUMAB
95	Nivolumab	IMM (Immune / cytokine)	NIVOLUMAB
96	Ipilimumab	IMM (Immune / cytokine)	IPILIMUMAB
97	Baclofen	CNS (Other CNS-active)	BACLOFEN
98	Topiramate	CNS (Other CNS-active)	TOPIRAMATE
99	Levetiracetam	CNS (Other CNS-active)	(?<!BRIVA)LEVETIRACETAM
100	Lamotrigine	CNS (Other CNS-active)	LAMOTRIGINE
101	Gabapentin	CNS (Other CNS-active)	GABAPENTIN
102	Pregabalin	CNS (Other CNS-active)	PREGABALIN
103	Zolpidem	CNS (Other CNS-active)	ZOLPIDEM
104	Varenicline	CNS (Other CNS-active)	VARENICLINE
105	Tramadol	CNS (Other CNS-active)	TRAMADOL
106	Sodium oxybate	CNS (Other CNS-active)	SODIUM OXYBATE|OXYBATE
107	Tetrabenazine	CNS (Other CNS-active)	TETRABENAZINE|VALBENAZINE|DEUTETRABENAZINE
108	Semaglutide	MET (Metabolic)	SEMAGLUTIDE
109	Liraglutide	MET (Metabolic)	LIRAGLUTIDE
110	Tirzepatide	MET (Metabolic)	TIRZEPATIDE
111	Dulaglutide	MET (Metabolic)	DULAGLUTIDE
112	Lithium	PSY (Psychotropic (indication comparator))	\bLITHIUM
113	Quetiapine	PSY (Psychotropic (indication comparator))	QUETIAPINE
114	Aripiprazole	PSY (Psychotropic (indication comparator))	(?<!BREX)ARIPIPRAZOLE
115	Olanzapine	PSY (Psychotropic (indication comparator))	OLANZAPINE
116	Risperidone	PSY (Psychotropic (indication comparator))	(?<!PALI)RISPERIDONE
117	Valproate	PSY (Psychotropic (indication comparator))	VALPROATE|VALPROIC|DIVALPROEX
118	Atorvastatin	CTRL (Negative control)	ATORVASTATIN
119	Amlodipine	CTRL (Negative control)	AMLODIPINE
120	Omeprazole	CTRL (Negative control)	(?<!ES)OMEPRAZOLE
121	Metformin	CTRL (Negative control)	METFORMIN
122	Losartan	CTRL (Negative control)	LOSARTAN
123	Levocetirizine	CTRL (Negative control)	LEVOCETIRIZINE|CETIRIZINE

Notation: "|" separates alternative spellings; "(?<!X)" is a negative lookbehind that prevents a match when the name is preceded by X (for example, CITALOPRAM is not matched within ESCITALOPRAM); "\b" marks a word boundary. Matching was performed on upper-cased text.

The same ingredient mapping dictionary, with every regular expression, is also provided in machine-readable form in Code S1 (drugs.py), where it is used directly by the analysis code.

S1.5 Indication and reporter variables

A case was flagged as carrying a bipolar-spectrum indication when any INDI record matched `BIPOLAR|MANIA|MANIC|CYCLOTHYM`, and as carrying any psychiatric indication when any INDI record additionally matched a broader psychiatric expression (depression, schizophrenia, psychosis, anxiety, obsessive–compulsive disorder, panic, post-traumatic stress, attention deficit, insomnia, substance or alcohol use). Reporter qualification was taken from the DEMO `occp_cod` field (MD physician, PH pharmacist, OT other health professional, CN consumer, LW lawyer).

S1.6 Time to onset

For each drug record matched as primary or secondary suspect, the interval between the therapy start date (THER `start_dt`, linked by drug sequence number) and the event date (DEMO `event_dt`) was computed. Partial dates were completed to the first day of the stated month or year. Intervals below zero or above 3,650 days were discarded. Both dates were available for 2,788 drug–report pairs among cases meeting the primary outcome definition. Because date completion could in principle create the latency ordering reported in the main text, a prespecified sensitivity analysis repeated the calculation using only records in which **both** the therapy start date and the event date were complete eight-digit dates (Supplementary Table S2). The ordering across mechanistic groups was preserved. Medians are reported by mechanistic group in Figure 5A and by agent in Supplementary Table S1, and are suppressed in Table 3 where fewer than five pairs were available. Because pooled medians give greater weight to drugs contributing many reports, latency was also summarized as the median of per-drug medians among drugs contributing at least five pairs (Supplementary Table S5).

S1.7 Disproportionality estimation

For each agent, a two-by-two table was formed of exposure (agent versus all other drugs) against outcome (mania or hypomania versus all other events). The reporting odds ratio was computed as *ad/bc* with a 95% confidence interval from the standard logarithmic error. The information component was computed as $IC = \log_2((a + 0.5)/(E + 0.5))$, where E is the expected count under independence, with the lower bound of its 95% credibility interval approximated as $IC_{025} = IC - 3.3(a + 0.5)^{-0.5} - 2(a + 0.5)^{-1.5}$. A signal was declared when the lower confidence bound of the reporting odds ratio exceeded 1, IC_{025} exceeded 0, and at least three cases were observed. No formal multiplicity adjustment was applied. The information component was used as a shrinkage-based complementary disproportionality measure to reduce instability in sparse drug–event combinations, which is not equivalent to a multiple-comparison correction. All analyses are descriptive and hypothesis-refining rather than confirmatory.

S1.8 Secondary and sensitivity analyses

Fourteen analyses were run in total: primary (PS, narrow outcome); suspect drugs (PS or SS, narrow outcome); PS with the broad outcome; exclusion of cases with a bipolar-spectrum indication; exclusion of cases with any psychiatric indication; four reporter-stratified analyses; two calendar-period analyses (2015–2020, 2021–2026); and three age-related analyses. Results for all 123 ingredients across the primary and principal sensitivity analyses (primary; suspect role; broad outcome; exclusion of bipolar-spectrum and of any psychiatric indication; physician- and consumer-reported; 2015–2020 and 2021–2026) are given in Supplementary Table S1; the remaining reporter-stratified and age-related analyses are reproducible with the code provided.

S1.9 Temporal-concentration flag

A raw single-year share cannot distinguish disproportionate manic reporting from a recently launched product whose entire report stream is recent. For each agent I therefore computed the share of its manic reports falling in its peak calendar year and divided it by the share of *all* that agent's reports falling in the same year. The ratio is proposed as a descriptive temporal-concentration flag, not as a validated diagnostic: it cannot separate litigation, batch submission, publicity, product launch and changing market share from one another, and it is unstable below roughly ten manic reports. In the present dataset duloxetine (mania share 0.623 versus all-report share 0.124 in 2017; ratio 5.0) and chloroquine (0.778 versus 0.157; ratio 5.0, based on only nine manic reports) have high ratios, whereas viloxazine (0.273 versus 0.292; ratio 0.9) and esketamine (0.311 versus 0.360; ratio 0.9) do not, despite raw single-year shares above 0.27. The flag is non-specific: among agents with at least ten manic reports, ropinirole (8.2) and two negative controls (amlodipine 6.7; levocetirizine 5.5) had higher ratios than duloxetine. Full results are given in Supplementary Table S3.

S1.10 Reporting standard and limitations

Reporting follows READUS-PV (references 49 and 50 of the main text). The analysis has no denominator and yields no incidence or risk estimate; exposure recorded as primary suspect reflects the reporter's causal belief; the outcome terms cannot capture subsyndromal activation; and estimates for agents with very large report streams dominated by other event types are subject to masking, which is a plausible but unestablished explanation for the inverse estimates observed for tumor necrosis factor-α inhibitors.

S1.11 Software and availability

Analyses were performed in Python 3.11 using pandas and NumPy. Generative artificial intelligence tools (Claude Opus 5, Anthropic) were used for English-language editing, code troubleshooting and data visualization. They were not used to select analytical outcomes or interpret the findings. All code was executed by the author and every reported value was verified by the author against the primary output files. The following files are provided (Code S1 comprises the files below; Table S1 is also provided as supplementary_table_S1.csv and Data S1 as time_to_onset.csv):

- `drugs.py` — ingredient dictionary, regular expressions, outcome and indication term sets
- `process.py` — per-quarter parsing of the FAERS ASCII extracts into a compact case table
- `analyze.py` — deduplication, disproportionality estimation, all sensitivity analyses, time-to-onset extraction
- faers_io.py — shared routines: project folders, reading of the quarterly tables and deletion files, and selection of the latest version of each case
- `make_outputs.py – construction of Table 3 of the main text and Table S1`
- `supplementary_table_S1.csv – results for all 123 ingredients across the primary and principal sensitivity analyses (Table S1)`
- `time_to_onset.csv` — Data S1: de-identified drug–interval pairs underlying Figure 5A
- `time_to_onset_completedates.csv – the complete-date-only sensitivity analysis (Table S2)`

- `temporal_concentration.csv` – the temporal-concentration flag for every agent (Table S3)
- `audit_sensitivity.py` – code for the complete-date latency analysis (Table S2) and the temporal-concentration flag (Table S3)
- `latency_druglevel.py` – pooled and drug-level latency summaries and the Kruskal–Wallis test (Table S5)
- `figures_pptx/lib.py, fig4.py, fig5.py` – Figures 4 and 5, drawn from the output files listed above (Figures 1–3 contain no computed values and were drawn directly)

The underlying FAERS quarterly files are public and are not redistributed here.
